# Obesity, but not high-fat diet, is associated with bone loss that is reversed via CD4^+^CD25^+^Foxp3^+^ Tregs-mediated gut microbiome of non-obese mice

**DOI:** 10.1038/s41538-023-00190-6

**Published:** 2023-04-13

**Authors:** Wei Song, Qinglin Sheng, Yuying Bai, Li Li, Xin Ning, Yangeng Liu, Chen Song, Tianyi Wang, Xiaohua Dong, Yane Luo, Jinhong Hu, Lina Zhu, Xiaole Cui, Bing Chen, Lingling Li, Congli Cai, Haobo Cui, Tianli Yue

**Affiliations:** 1 College of Food Science and Technology, Northwest University, 710069 Xi’an, China; 2 Laboratory of Nutritional and Healthy Food-Individuation Manufacturing Engineering, 710069 Xi’an, China; 3 Research Center of Food Safety Risk Assessment and Control, 710069 Xi’an, China; 4 School of Life Science and Technology, Tokyo Institute of Technology, 226-8501 Yokohama, Japan; 5 Department of Food Science and Technology, Harbin Institute of Technology, 150000 Harbin, China; 6 National Local Joint Laboratory of Extreme Environmental Nutritional Molecule Synthesis Transformation and Separation, 150000 Harbin, China

**Keywords:** Immunological disorders, Weight management, Microbiota, Microbiome

## Abstract

Osteoporosis is characterized by decreased bone mass, microarchitectural deterioration, and increased bone fragility. High-fat diet (HFD)-induced obesity also results in bone loss, which is associated with an imbalanced gut microbiome. However, whether HFD-induced obesity or HFD itself promotes osteoclastogenesis and consequent bone loss remains unclear. In this study, we developed HFD-induced obesity (HIO) and non-obesity (NO) mouse models to evaluate the effect of HFD on bone loss. NO mice were defined as body weight within 5% of higher or lower than that of chow diet fed mice after 10 weeks HFD feeding. NO was protected from HIO-induced bone loss by the RANKL /OPG system, with associated increases in the tibia tenacity, cortical bone mean density, bone volume of cancellous bone, and trabecular number. This led to increased bone strength and improved bone microstructure via the microbiome-short-chain fatty acids (SCFAs) regulation. Additionally, endogenous gut-SCFAs produced by the NO mice activated free fatty acid receptor 2 and inhibited histone deacetylases, resulting in the promotion of Treg cell proliferation in the HFD-fed NO mice; thereby, inhibiting osteoclastogenesis, which can be transplanted by fecal microbiome. Furthermore, T cells from NO mice retain differentiation of osteoclast precursors of RAW 264.7 macrophages ex vivo. Our data reveal that HFD is not a deleterious diet; however, the induction of obesity serves as a key trigger of bone loss that can be blocked by a NO mouse-specific gut microbiome.

## Introduction

Osteoporosis, characterized by decreased bone mass, microarchitectural deterioration and increased bone fragility, is the most prevalent chronic metabolic bone disease, subsequent increases in the risk of fracture^1–3^. Bone loss results from an imbalance in bone remodeling^4–7^. Although there are many preventive methods and treatments for bone loss, their application requires exertion by patients, who may limit their physical exercise, or includes unwanted side effects and drug costs, such as hormone therapies. These drawbacks decrease their utilization and increase the risk of bone loss. Therefore, diet intervention is considered the safest and most easily implemented treatment to improve bone health and reduce bone loss^8–12^.

Abnormal bone metabolism and resorption are influenced by many factors, such as genetics, hormones, age, and nutrition, among which dietary factors play an important role^13–15^. A high-fat diet (HFD) has become a common dietary pattern worldwide^16–18^. Recent studies suggest that an HFD not only leads to obesity but also induces metabolic abnormalities and absorption of bone, resulting in reduced bone mass and low bone strength, thus increasing the risk of spontaneous and traumatic bone injury^19–22^. This suggests that excess fat is detrimental to bone health^23–25^. However, while it has been considered a poor dietary choice, an HFD does not invariably lead to obesity^26–28^. Moreover, non-obese individuals with an HFD and their associated bone health have barely been studied, which raises the question as to whether HFD-induced obesity or an HFD alone leads to bone loss.

Gut microbiota, known as the second gene pool of the human body, plays a critical role in regulating host physiology, including bone remodeling in animal subjects and human patients^29–31^. Intestinal microbiomes mediate bone metabolism by regulating immune status, endocrine coordination, flora composition, and their generated metabolites^32–34^. Short-chain fatty acids (SCFAs), the main product of human bacterial fermentation, enhance body weight density and regulate osteoclastogenesis to prevent bone loss, which is an effective regulator of osteoclast (OC) metabolism and bone homeostasis^35–38^. Furthermore, SCFAs regulate the number and function of colonic regulatory T cells (Tregs) through inhibiting histone deacetylases (HDACs) and activating the G-protein-coupled receptor signaling cascade in mice^39–41^. Tregs have been shown to ameliorate arthritis and bone loss in autoimmune animal models and ovariectomized rats, respectively^42–44^. However, how they are involved in bone loss in HFD-induced obese mice is unclear.

In the present study, we show that HFD-induced obesity leads to gut dysbiosis and consequent bone loss. Furthermore, using biomechanical and structural bone parameters, we demonstrate that HFD-fed non-obese (NO) mice still build up healthy bones despite long-term HFD. These osteological improvements were mediated by gut microbiome-SCFA-regulation and were transferrable to obese mice through fecal microbiota transplantation (FMT). Mechanistically, we show that endogenous gut SCFA-mediated Tregs and HDACs are critical for maintaining bone health through the gut microbiome-bone axis.

## Results

### HFD-induced obesity, but not HFD alone, leads to bone loss

**Fig. 1:**
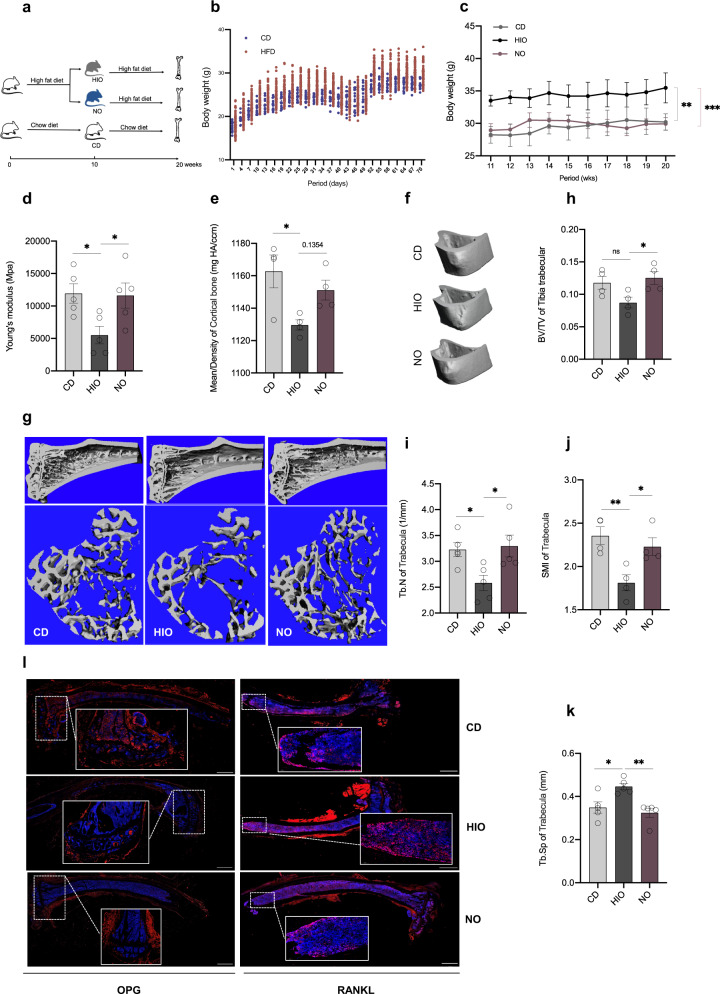
HIO, but not HFD, induced bone loss in mice. **a** Study design. Tibia were harvest after 20 weeks HFD intervene for HIO and NO mice on bone property analysis. **b** Body weight changes after chow diet (*n* = 14) and HFD (*n* = 81) fed during 70 days. **c** Body weight of HIO, NO, and CD mice (*n* = 7) after 10 weeks of HFD or chow diet, respectively. **d** Young’s modulus (*n* = 5). Tibia was analyzed by μ-CT (*n* = 4–6) to access (**e**) the mean/density of cortical bone, (**f**) generate representative cortical bone images, (**g**) three-dimensional reconstruction images of cancellous bone, (**h**) BV/TV of tibia trabecular, (**i**) trabecular number (Tb. N), (**j**) SIM of Trabecula and (**k**) trabecular separation/spacing (Tb. Sp). **l** Immunohistochemistry of OPG and RANKL expression (12×), length of the scale bar is 1000 μm. Data are presented as means ± standard errors of the means (± SEM). Results determined by one-way ANOVA. ^*^p < 0.05, ^**^p < 0.01, ^***^p < 0.001.

To address whether HFD alone or HFD-induced obesity contribute to the observed bone loss, HIO mice were defined as having body weight higher than 20% of chow diet (CD) mice, while NO mice were defined as body weight within 5% of the CD weight range after 10 weeks of HFD feeding. After that, NO and HIO mice were fed HFD for another 10 weeks. NO mice receiving an extended HFD had a stable weight, but significantly lower serum triglycerides (TG) and low-density lipoprotein (LDL) compared to HIO mice during these later 10 weeks (Fig. 1a–c and S1a–d) followed by reduced numbers and smaller sizes of fat vacuoles in hepatocytes and white adipose tissues (Fig. S1e–g). This was accompanied by a trend toward a significant increased Young’s modulus (Fig. 1d), suggesting preferred tenacity of the tibia, with no significant changes in maximum bending stress and maximum bending load (Fig. S1h, i) in a three-point bending test.

Biomechanical changes were also reflected at a structural level. HIO mice were significantly different from CD mice, while NO mice were similar. However, while the decrease in mean/density of cortical bone was enhanced in NO compared to HIO mice (Fig. 1e, f), it was not significantly different and there were no effects on cortical thickness (Ct.Th) (Fig. S1j). As an extension of cortical bone into cancellous bone, the quality of trabecular bone is closely related to its microstructure. The μ-CT and three-dimensional reconstruction images (Fig. 1g) showed that HIO had reduced bone volume to tissue volume (BV/TV) ratio of tibia trabecular, while NO had markedly elevated the value (Fig. 1h). A robust reduction in trabecular bone (Tb.N) (Fig. 1i) and structure model index (SMI) (Fig. 1j) and increases in trabecular separation (Tb.Sp) (Fig. 1k) were observed in HIO mice, whereas the NO group reversed these changes. Based on the above findings, we investigated whether osteoclastogenesis was involved. By immunostaining tibia, we determined substantially higher osteoprotegerin (OPG) and lower receptor activator of nuclear factor-κB ligand (RANKL) expression in NO mice, suggesting inhibition of OC differentiation compared with the HIO group (Fig. 1l). RANKL, as a type II transmembrane protein, is the only factor found to induce osteoclast differentiation and development by combined with receptor activator of nuclear factor-κB (RANK). Moreover, OPG and RANKL competitive combination with RANK to inhibit bone resorption and promote bone formation^45^. These data show that NO mice retained normal biomechanical and structural bone parameters despite a long-term HFD, unlike the HIO mice.

### The gut microbiome determines the differences between obese and non-obese mice under HFD

Next, we investigate why the same HFD led to different levels of weight gain with entirely different bone qualities. Recent evidence suggests an interaction between gut microbiota and bone metabolism^5^. We, therefore, investigated whether microbiota compositions were altered with HFD in NO versus HIO mice. As shown by principal component analysis (PCA), the NO group was closer to the CD group than to the HIO group on PC1 which explained 59.77% of the total variation, indicating that the gut microbiota composition of NO was similar to the CD group (Fig. S2a).

**Fig. 2:**
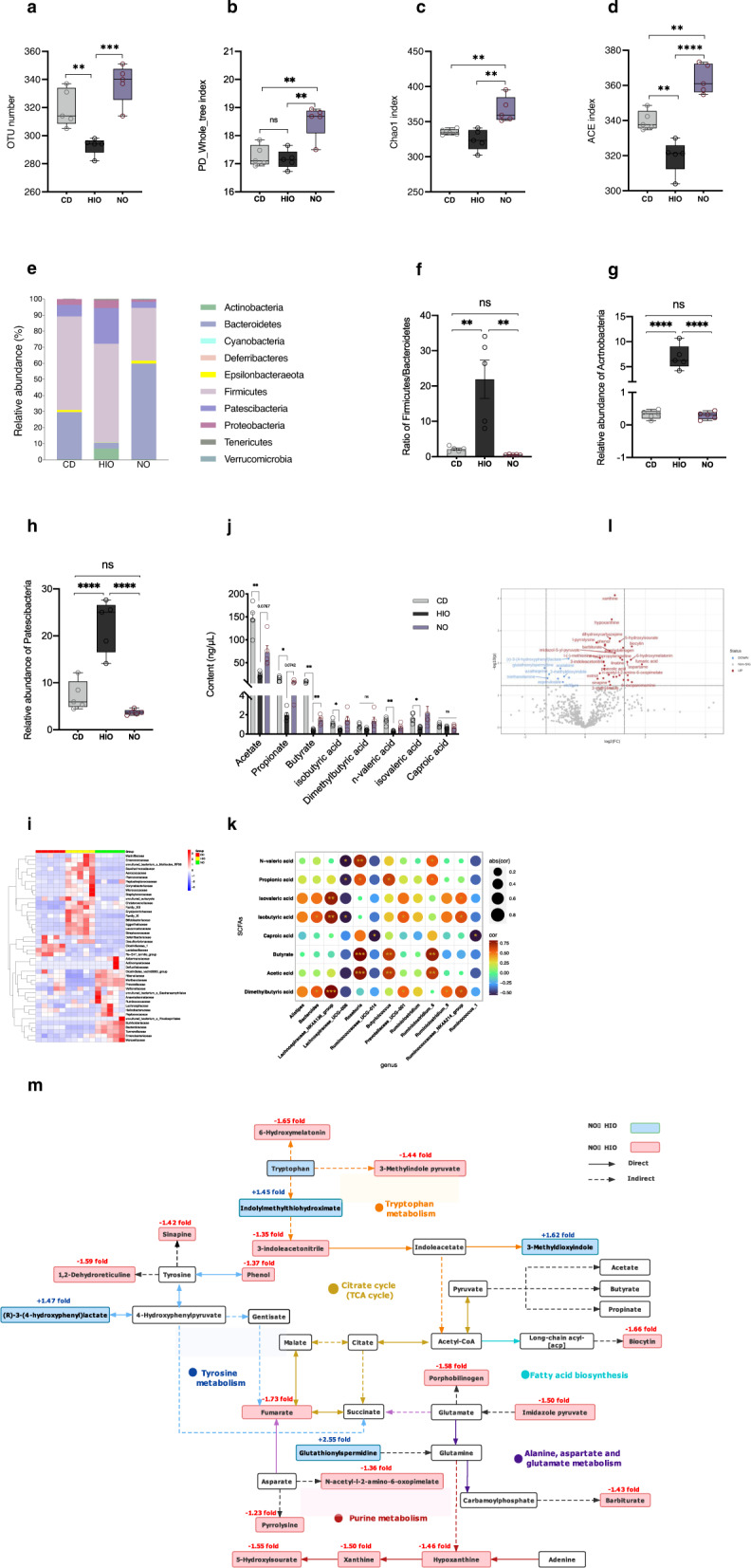
Gut microbiome prevents occurrence of obesity from HFD in NO mice. **a**–**d** OTU number, PD_Whole_tree, chao1 and ACE indices of alpha diversity. **e** Relative abundance of phyla. **f** Ratio of *Firmicutes*/*Bacteroidetes*. **g**, **h** Relative abundance of *Acrtnobacteria* and *Patescibacteria*. **i** Heat map representing differentially abundant taxa (genus with higher hierarchy family name) in CD, HIO and NO groups, which has been normalized to the range of −4 to 4. **j** Fecal SCFAs content composed of acetate, propionate, butyrate and isobutyrate, dimethylbutyric acid, n-valeric acid, isovaleric acid, and caproic acid. **k** Correlations between kinds of genus and eight types of SCFAs in CD, HIO and NO groups. **l** Volcano plot showing differentially expressed metabolites in feces from HIO and NO mice; red dots indicate upregulated in HIO group, blue dots indicate downregulated in HIO group. **m** Summary of metabolic pathways which include discriminating metabolites. Green rectangle—significant metabolites with an increased fold change in NO vs. HIO comparison. Red rectangle—significant metabolites with an decreased fold change in NO vs. HIO comparison. *n* = 5 per group for high-throughput sequencing of the 16S rDNA gene of fecal bacteria; *n* = 5–7 per group for Fecal metabolomics. Correlations were performed using Pearson test by “R” packet. Two-way ANOVA was used for SCFAs content analysis of (**j**). Data are presented as means ± SEM. One-way ANOVA was used to analyze significant differences in the rest of the data. ^*^p < 0.05, ^**^p < 0.01, ^***^p < 0.001, ^****^p < 0.0001.

Alpha analysis showed that the operational taxonomic unit (OTU) number and PD_Whole_tree index were increased in NO compared to HIO mice, indicating greater diversity of bacterial species (Fig. 2a, b). In addition, the Chao1 and ACE indices, which take into account species abundance, were increased in NO mice (Fig. 2c, d). In contrast to the HIO group, the ratio of Firmicutes to Bacteroidetes was markedly decreased in the NO group, suggesting that the change in the ratio was related to the development of obesity^46^ (Fig. 2e, f). Notably, bacterial differential abundance at the phylum level of Actinobacteria and Patescibacteria in NO were significantly lower compared to the HIO group, which were previously shown to be increased in obese patients and in the adipose tissue of obese people^47,48^, respectively (Fig. 2g, h). The family taxa heat map (Fig. 2i) revealed an HIO microbiota signature associated with an increase in families of *Erysipelotrichaceae*, *Enterococcaceae*, *Peptostreptococcaceae* and *Corynebacteriaceae* (Fig. S2b) which are negatively related with obesity^49–52^. HIO microbiota were also associated with increases in disease-associated families *Saccharimonadaceae*, *Aerococcaceae*, and *Eggerthellaceae* (Fig. S2c), which are related to genetic risk for autoimmunity, schizophrenia and depression, respectively^53–55^. NO microbial composition was, however, different than the HIO microbial composition, as well as CD one, with overall increases in SCFA-producing bacteria *Akkemansiaceae*, *Prevotellaceae*, *Ruminococcaceae*, *Lachnospiraceae, Bacteroidaceae* and *Enterobacteriaceae*^56–58^, and obesity negative-related bacteria *Rikenellaceae* and *Muribaculaceae* (Fig. S2d)^26,59,60^, suggesting that the NO gut environment favored the selective expansion of SCFA-producing or obesity negative-related bacterial taxa.

To confirm that changes in gut microbiota composition correlated with SCFAs, we analyzed the fecal SCFA content and its correlation with the genera identified. There was little difference between CD and NO mice, while as expected, HIO mice differed for most SCFAs. Strikingly, NO had increased acetate and butyrate content compared to that of HIO mice. NO mice also had increased propionate content but it was not significant (Fig. 2j). Furthermore, correlation analysis showed that the genera *Alistipes*, *Bacteroides*, *Lachnospiraceae_NK4A136_group*, *prevotellaceae*_UCG-001, *Ruminiclostridium_9* and *Ruminococcaceae_NK4A214_group* were positively correlated with isovaleric acid, isobutyric acid or dimethybutyric acid (Fig. 2k). Consistently, the relative abundance of those genera was over-represented in NO mice (Fig. S2e). Significant correlations were also observed between *Roseburia*, *Butyricicoccus* and *Ruminiclostridium_6* and butyrate or acetic acid content. NO mice exhibited lower relative abundance of *Lachnospiraceae_UCG-006* (Fig. S2f) than HIO mice, which was negatively correlated with N-valeric acid, propionic acid, isobutyric acid, caproic acid, butyrate or acetic acid (Fig. 2k). These data suggest that NO mice resist HFD-induced obesity through increased SCFA-producing bacteria in their gut.

As critical regulators, gut microbiota should change other metabolites in addition to SCFA content. To reveal the metabolic phenotype related to the gut microbiome, which could potentially explain why the same HFD results in different body weights, we performed metabolomic profiling of the feces of HIO and NO mice. Through comparison of the two groups, 50 fecal metabolites were identified with significant peaks in negative and positive ionization modes (Fig. 2l and S2g, h). Analysis using the Kyoto Encyclopedia of Genes and Genomes (KEGG) showed that enrichment of metabolites in NO pathways primarily involved phenylalanine, tyrosine and tryptophan biosynthesis; phenylalanine metabolism; alanine, aspartate, and glutamate metabolism; and pyruvate metabolism as compared to HIO (Fig. S2i). In the integrated metabolic pathways ((Fig. 2m), tryptophan catabolic fluxes were enhanced in the HIO gut, as characterized by increased levels of 6-hydroxymelatonin (−1.65-fold), 3-methylindole pyruvate (−1.44-fold), and 3-indoleacetonitrile (−1.35-fold), and decreased levels of indolylmethylthiohydroximate (+1.45-fold), and 3-methyldioxyindole (+1.62-old) (Fig. S2j). Furthermore, increased levels of tyrosine metabolite of sinapine (−1.42 fold), 1,2-dehydroreticuline (−1.59 fold), phenol (−1.37 fold) and fumarate (−1.73 fold) were shown in HIO mouse feces (Fig. S2j). We also observed that all three purine metabolite levels including 5-hydroxyisourate (−1.55 fold), xanthine (−1.50-fold), and hypoxanthine (−1.46-fold) were reduced in NO mice (Fig. S2j), which was negatively correlated with the bone parameters of SMI, Tb.N, BV/TV, bone mean/density and Young’s modulus, but positively correlated with Tb.sp (Fig. S2k).

### SCFA treatment ameliorates HFD obesity-induced bone loss

**Fig. 3:**
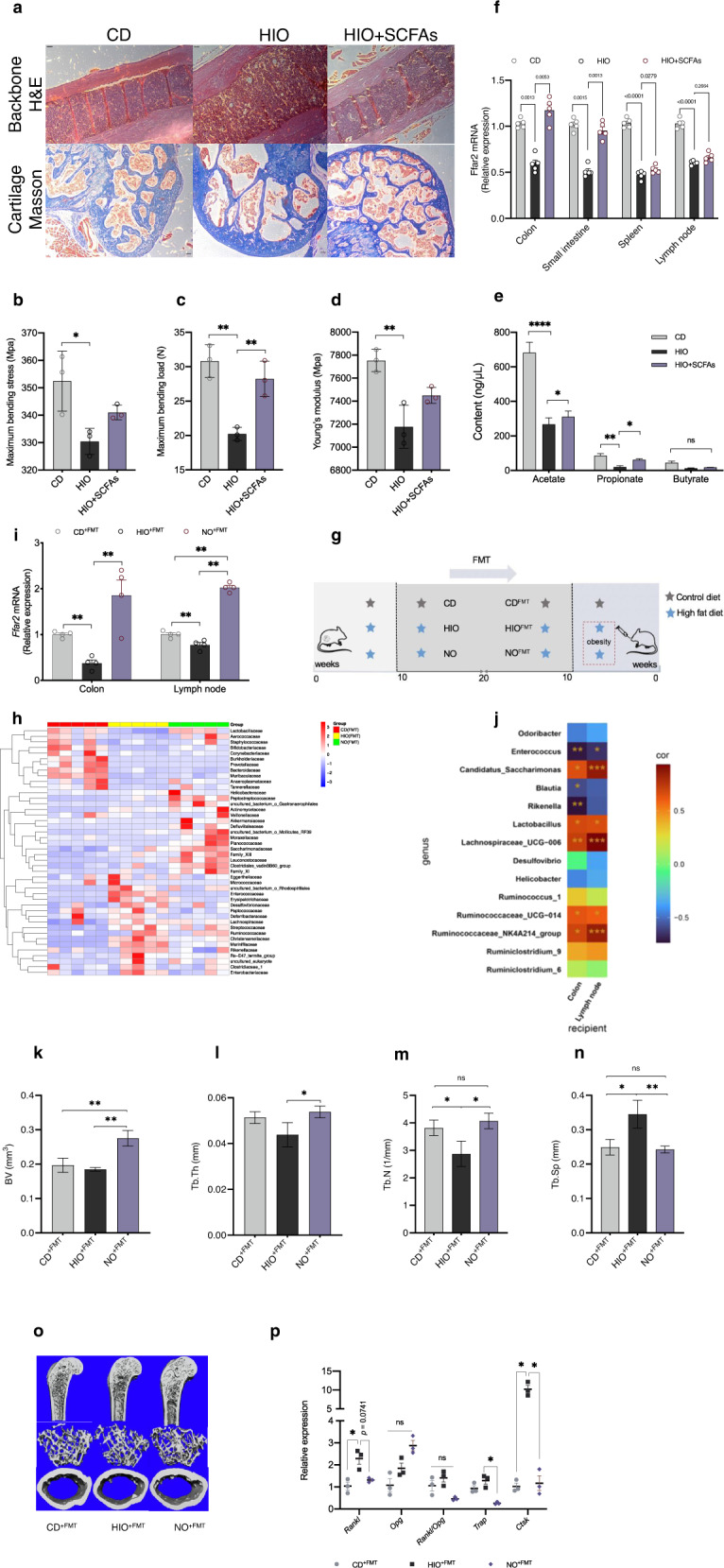
SCFAs contribute to reversed femur bone loss in HFD-induced NO mice. **a** H&E staining of backbone and masson trichrome staining (100×), length of the scale bar is 500 μm. **b** Maximum bending stress. **c** Maximum bending load. **d** Young’s modulus (*n* = 3). **e** Fecal SCFAs content composed of acetate, propionate and butyrate (*n* = 5). **f** Relative Ffar2 mRNA expression in colon, small intestine, spleen, and lymph node (*n* = 5). **g** Study design. Five-week-old HFD-fed mice were colonized with feces from the indicated mice for 10 weeks. **h** Heat map of the selected most differentially abundant features at the family level. **i** Relative *Ffar2* mRNA expression in colon and lymph (*n* = 4). **j** Correlations between two different locations of recipients and kinds of genus. Correlations were performed using Pearson test between two different locations of recipients and kinds of genus by “R” packet. **k**–**o** μ-CT was conducted to determine the bone parameters of BV, Tb. Th, Tb. N, Tb. Sp, and obtain 3D images (*n* = 3). **p** Relative mRNA expression of *Rankl*, *Opg*, *Rankl*/*Opg*, *Trap* and *Ctsk* (*n* = 3–4). Data are presented as means ± SEM. Results determined by one-way ANOVA for (**b**–**d**) and (**k**–**n**), and two-way ANOVA for (**e**, **f**, **i**, **p**). ^*^p < 0.05, ^**^p < 0.01, ^****^p < 0.0001.

To confirm whether microbiota impacts the bone parameters through SCFA production, we first administrated a SCFA mix intragastrically to HIO mice. SCFAs improved the trabecular meshwork at the femoral head with relatively increased reticular formation and a neatly arranged and complete microstructure. In addition, cartilage collagen fibers also recovered from a disordered loose and dissolved arrangement to become more matured (Fig. 3a). These structural improvements were accompanied by reduced fragility of bones in SCFA-treated mice, as shown by the biomechanical measurements during the three-point test (Fig. 3b–d). The SCFA mix also elevated acetate and propionate levels in feces, which were mainly produced by the applicable gut microbiota (Fig. 3e). Correspondingly, SCFA gavage significantly enhanced free fatty acid receptor 2 (Ffar2) expression which can be activated by SCFA in the colon, small intestine, and spleen (Fig. 3f). To directly elucidate whether SCFAs were involved in the prevention of bone loss in NO mice through regulation of SCFA-producing gut microbiota, we transplanted the fecal microbiota (FMT) from NO mice into HIO mice (Fig. 3g), while maintaining the diet (HFD for HIO, NO, HIO^+FMT^ and NO^+FMT^ mice; chow for CD and CD^+FMT^ control mice). Alpha-diversity analysis showed higher ACE, Chao1, and PD_Whole_tree indices in NO^+FMT^ mice after the transplantation than those of the CD^+FMT^ and HIO^+FMT^ groups (Fig. S3a). Interestingly, alpha diversity was elevated after FMT in all groups compared to pretransplantation. At the family level, the heat map showed *Eggerthellaceae*, *Enterococcaceae* and *Erysipelotrichaceae* were more abundant in the HIO^+FMT^ group than in the CD^+FMT^ and NO^+FMT^ groups (Fig. 3h and S3b); in addition, NO^+FMT^ mice had increased the abundance of *Akkemansiaceae*, which was partly in accordance with the results obtained before the transplantation (Fig. 3h and S3c; Fig. S2b–d). *Ffar2* mRNA expression in colon and lymph node tissue was significantly increased after NO mice fecal transplantation and showed positive correlation with *Candidatus_Saccharimonas*, *Lactobacillus*, *Lachnospiraceae_*UCG-006, *Ruminococcaceae_*UCG-014, and *Ruminococcaceae_*NK4A214_group; and negative correlation with *Enterococcus*, *Blautia* and *Rikenella* (Fig. 3i, j and S3d). Together, these data demonstrated that SCFAs produced by the gut microbiome were involved in inhibition of bone loss by NO.

To uncover whether the transplanted microbiota enhanced bone strength, we also performed a similar analysis for parameters of bone tissue. Fecal transplantation of NO microbiota significantly increased the values of BV, Tb.Th, and Tb. N, but decreased Tb.Sp values in HIO^+FMT^ mice, which was consistent with the μ-CT bone imaging results, suggesting that any femur bone loss had been substantially alleviated (Fig. 3k–o). In contrast, the mean/density and BV/TV values remained unchanged (Fig. S3e). The enhancement of gene expression of *Trap* and *Ctsk* was correspondingly decreased with fecal transplantation of NO mice (Fig. 3p). Furthermore, the serum content of RANKL, but not TRAP, in NO^+FMT^ mice was significantly lower than in HIO^+FMT^ mice (Fig. S3f). Thus, HIO mice transplanted with NO gut microbiota acquired SCFA-producing microbiome that efficiently ameliorated bone loss from HFD-induced obesity.

### NO gut microbiome affects CD4^+^CD25^+^Foxp3^+^ Treg proliferation

**Fig. 4:**
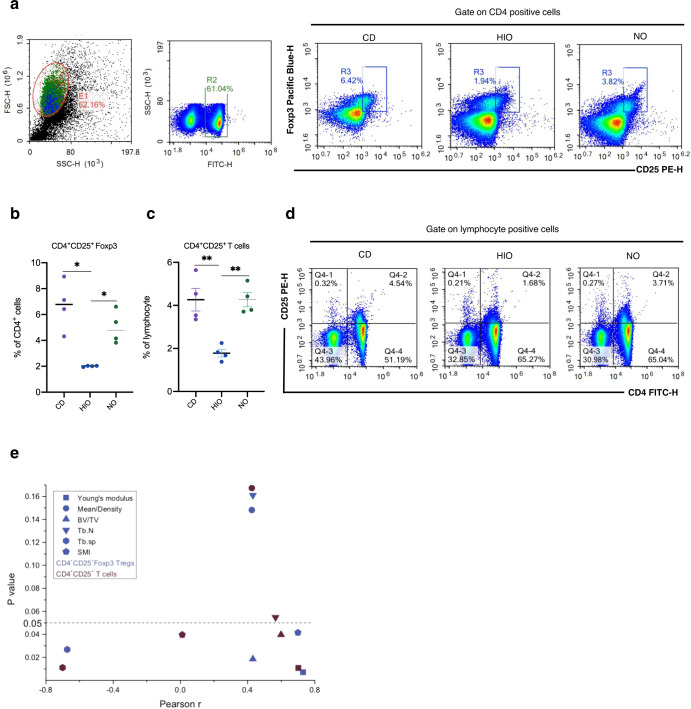
NO regulates Treg homeostasis. **a** CD4^+^CD25^+^ Foxp3 Treg population from mesenteric lymph nodes. **b** Percentage of CD4^+^CD25^+^ Foxp3 Tregs. **c** Percentage of CD4^+^CD25^+^ T cell. **d** CD4^+^CD25^+^ T cells population. **e** Correlation between CD4^+^CD25^+^ Foxp3 Tregs and CD4^+^CD25^+^ T cells against the bone parameters of Young’s modules, Mean/Density of cortical bone, BV/TV of cancellous, Tb.N of trabecula, Tb.sp of trabecula and SMI of trabecula. *n* = 4 mice per group. Correlations were performed using Pearson test between two different locations of recipients and kinds of genus by “R” packet. Data are presented as means ± SEM. One-way ANOVA was used to analyze significant differences. ^*^p < 0.05, ^**^p < 0.01.

SCFAs can regulate the number and function of colonic Tregs in mice. Recent studies have shown a close relationship between immune cells and bone cells, which share regulatory molecules such as cytokines and their receptors, signaling molecules, and transcription factors^41,61,62^. The above results indicated that the lack of obesity in NO mice was modulated via SCFAs from the gut microbiota, resulting in significant modulation of OC differentiation. The next set of measurements were made to determine the involvement of Treg-related factors. Our results showed a dramatically higher percentage of both CD4^+^CD25^+^Foxp3^+^ Tregs and CD4^+^CD25^+^ T cells in the NO mice compared with those in the HIO mice (Fig. 4a–d). Expectedly, Young’s modulus, BV/TV of cancellous bone, and SMI of trabecular bone showed a significant positive correlation with CD4^+^CD25^+^ Foxp3^+^ Tregs and CD4^+^CD25^+^ T cells (Fig. 4e and S4a–f). However, Tb.sp of trabecula was negatively correlated with CD4^+^CD25^+^ Foxp3^+^ Tregs and CD4^+^CD25^+^ T cells (Fig. S4g, h). No correlation was found between mean/density of cortical bone or Tb.N of trabecula and the above two parameters. (Fig. 4e). Tregs that are characterized as expression of forkhead box p3 (*Foxp3)* secretes cytokines interleukin 10 (*Il10)* and transforming growth factor *(Tgf-β1)* can regulate OC differentiation^41^. We further demonstrated that gavaged SCFAs enhanced *Foxp3* and *Il10* but not *Tgf-β1* mRNA expression in the colon and small intestine (Fig. S4i–k), which supports our hypothesis that SCFAs were involved in Tregs regulation by NO.

**Fig. 5:**
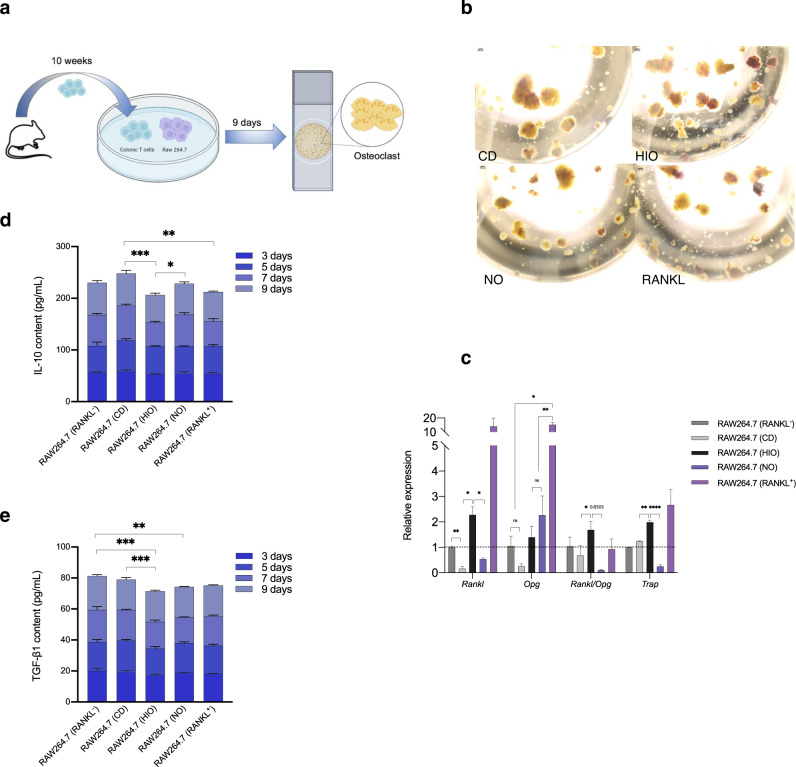
T cells inhibit differentiation of RAW 264.7 macrophages ex vivo. **a** Study design. T cells obtained from CD, HIO, and NO were cocultured with RAW264.7 macrophages for 9 days. **b** TRAP staining. **c** Total RNA was extracted from the RAW264.7 macrophages, and the relative expression of *Rankl*, *Opg*, *Rankl*/*Opg* and *Trap* was determined. **d**–**e** Assessment of cytokines of IL-10 and TGF-β levels in cell supernatants. *n* = 3 per group. Data are presented as means ± SEM. Two-way ANOVA was used to analyze the significance of differences. ^*^p < 0.05, ^**^p < 0.01, ^***^p < 0.001, ^****^p < 0.0001.

To confirm the effects of Tregs on bone cellular activity, we performed an ex vivo experiment, using T cells from spleen cocultured with the murine mononuclear macrophages RAW 264.7 cells (Fig. 5a). We found that T cells obtained from NO mice inhibited the differentiation of RAW 264.7 macrophages, as indicated by a reduction in purple reaction and reduced numbers of multinucleated cells after 10 days of direct coculture (Fig. 5b). T cells extracted from NO mice significantly downregulated the relative RNA expression of *Rankl* and *Trap* in RAW 264.7 macrophages. Furthermore, substantial down-regulation of the ratio of *Rankl/Opg* was observed in the NO group (Fig. 5c). Additionally, supernatants obtained from cultured cells showed significant higher levels of IL-10 in the NO cultures during 9 days coculture compared with those of HIO mice, however, the TGF-β1 level was not significance (Fig. 5d, e).

**Fig. 6:**
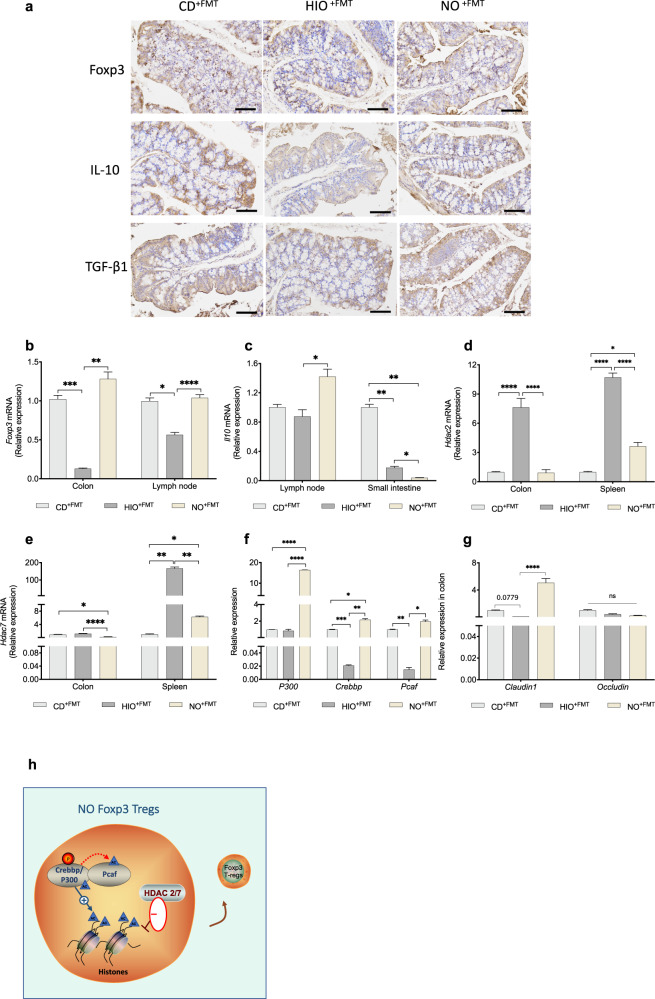
NO mice fecal transplantation regulates Treg homeostasis. **a** Transplanted fecal material enhanced the expression of the colon Treg-related immune factors Foxp3 and IL-10 and TGF-β1, as measured by immunohistochemistry (400×), length of the scale bar is 200 μm. **b**, **c** Relative mRNA expression levels of *Foxp3* and *Il10* measured in the colon, lymph nodes, or small intestine. **d**, **e** Relative mRNA expression levels of *Hdacs*, including *Hdac2* and *Hdac7* measured in the colon and spleen. **f** Expression of histone acetyltransferases, including *P300*, *Crebbp* and *Pcaf*, in the colon. **g** *Claudin1* and *Occludin* mRNA expression in the colon. **h** Figure abstract of the regulatory mechanism of acetylated Foxp3. Two-way ANOVA was used to analyze the significance of differences in the relative mRNA expression. Data are presented as means ± SEM (*n* = 3). ^*^p < 0.05, ^**^p < 0.01, ^***^p < 0.001, ^****^p < 0.0001.

To confirm whether the effect on CD4^+^CD25^+^ Foxp3^+^ Tregs was caused by the transplanted microbiota, we measured Treg-relative factors in recipient mice. The recurrent microbiota transplantation of NO microbiota significantly promoted the proliferation of Foxp3^+^ Tregs in the colon tissue of mice and increased the expression of IL-10 (brown-yellow spots in Fig. 6a), suggesting increased intestinal immunoregulation that could inhibit OC differentiation. We also examined the relative gene expression of *Foxp3*, *IL-10*, and *TGF-β1* in the systemic immune system. *Foxp3* expression in the NO^+FMT^ groups was markedly increased in the lymph nodes and colon (Fig. 6b), and the levels of *Il10*, a marker for bone OC differentiation, were increased in the lymph nodes (Fig. 6c). Of note, TGF-β1 showed no significant change (Fig. S4l). Overall, the Tregs immune response via IL-10 related to NO gut microbiota transplantation not only affected the function of the colon and small intestine but also influenced the more distant organ of lymphatic tissues of the immune system.

The expression of *Hdac-2* and *Hdac-7* in the colon and spleen of NO^+FMT^ mice was significantly downregulated (Fig. 6d, e). In the colon, NO mice fecal microbiota transfer upregulated the expression of the acetylation-related genes *P300*, *Crebbp*, and *Pcaf* (Fig. 6f). *P300* is an important acetylation-related gene that functions in the colon and small intestine. The interaction between P300 and Foxp3 can promote the acetylation of Foxp3 (Fig. 6h). Acetylated Foxp3 enhances the stability of Foxp3 and promotes the normal functioning of Tregs in the colon^63–65^. The relative expression of the *Foxp3* gene in the colon and small intestine observed in our present study is also consistent with this. Furthermore, NO FMT recovered HIO-associated intestinal barrier damage by elevating *Claudin1* mRNA expression (Fig. 6g).

## Discussion

Excessive fat intake causes obesity and microinflammation, resulting in bone fragility and bone loss^66^^,^^67^. Although the relationship between obesity and bone loss is controversial, some studies have found that under HFD stimulation, with the occurrence of obesity, bone metabolism occurs in the femur and induces abnormalities in bone structure and bone strength^20,24^. However, whether HFD alone preferentially drives bone loss with or without obesity and by what mechanism, other than a simple causal relationship, has not been studied. Our data show that physical and biomechanical properties of bone are largely affected by the obesity itself, but not by the HFD, and that HFD-fed NO mice had normal trabecular and cancellous bone structure and strength. To our knowledge, these datasets clarify the distinction between HFD-induced obese mice and HFD-fed NO mice regarding bone properties for the first time. Interestingly, NO mice did not have the negative effects on bone that HFD-induced obese (HIO) mice had. Many people who have high-fat and high-caloric food are still slim and healthy along with consequently healthy bones^68–70^. There could be several explanations regarding the origin of these individual differences, including gut microbiota, and it has been proposed that genetics and metabolic factors as well as ethnic factors, play significant roles^71–74^. Without excluding any of the above-mentioned causes, our data analysis supports the notion that compensatory gut microbiota prevents obesity and gut dysbiosis^75,76^ suggesting that HFD-independent effects of bone loss due to HFD-induced obesity itself can be ameliorated through altering gut microbiota based on the NO type assemblage. In addition, our work shows that HFD-induced bone loss could be alleviated through FMT of NO microbiota, suggesting a major protective effect for the overall microbiota.

Studies have shown that SCFAs are regulators of OC metabolism and bone mass in vivo^36,77^. Of note, further studies indicate that SCFA-producing bacteria and the SCFA metabolic products, specifically, acetate propionate and butyrate found in the NO group, are involved in gut microbiota modulation. Therefore, we applied a SCFA mix and confirmed that it exerts bone protective effects in HIO mice. Consistent with this finding, our results found that transplanted fecal microbiota from NO mice enhanced bone strength and microstructure, as well as decreased relevant osteoclast genes, by altering the microbiome, which was positively correlated with *Ffar2*. We also identified purines including 5-hydroxyisourate, xanthine and hypoxanthine, as obesity-related metabolites that may reflect bone health. Excessive purines can lead to hyperuricemia, which is a known factor for gouty arthritis^78–80^. The gut microbiota is involved in purine oxidative metabolism by secreting active enzymes^81,82^. However, increases in SCFA levels can inhibit xanthine oxidase activity and finally alleviate hyperuricemia^83,84^. Hence, our results suggested that the NO microbiome might exert its bone-protective effects through SCFAs produced by their reshaped microbiota.

SCFAs have the ability to influence Tregs development and promote bone metabolism by inhibiting the occurrence, differentiation, and function of OCs, thus blocking bone destruction^85,86^. Ffar2, as one of the major SCFA receptors, has been shown to combine with SCFAs to promote Treg development and impede the progression of colitis^87,88^. SCFAs alter gene expression by regulating the activity of *Hdacs* as well as the histone acetyltransferase *P300* through epigenetic mechanisms^87,88^. The interaction between Foxp3 and P300 induces Foxp3 acetylation to enhance *Foxp3* stability, thus boosting Treg immune function^89,90^. Notably, our work shows that after NO^+FMT^, the expression of *Hdac2* and *Hdac7* was decreased, while the expression of *Ffar2*, *P300*, *Crebbp* and *Pcaf* was increased, which contributed to SCFA-induced Treg development. *Foxp3* expression, as well as *Il10* expression, increased not only in the colon, but also in the lymph nodes, suggesting that systemic immunoregulation was involved in this regulation. Systemic Treg regulation facilitated Foxp3^+^ Treg proliferation and the production of the Treg factor IL-10, which inhibited OC differentiation.

In summary, this study indicates that HFD-indued obesity and not HFD itself leads to bone fragility and bone loss through homeostasis disequilibrium of the gut microbiota. SCFAs produced by gut bacteria, as significant metabolites, can affect HDACs directly or through activating Ffar2 to regulate IL-10 Tregs function, leading to balanced maintenance of osteoclastogenesis via the gut-bone axis in NO mice despite the HFD. Enhanced gut SCFAs may serve to explain the bone quality differences between obese and NO individuals after HFD and is a promising strategy to address bone loss with obesity. These findings and insights suggest that whether or not an HFD is good or bad diet for an individual is not decided by the dietary ingredients but by the individual’s gut microbiome, which may prove valuable for precisely tailoring nutrition strategies in the future.

## Methods

### Animal model

Animal experimental procedures followed the National Institutes of Health guidelines for the care and use of laboratory animals and were approved by the ethics committee of the 2nd Affiliated Hospital of Harbin Medical University (Approval No. SYXK-(HEI) 2019-001). Five-week-old, specific pathogen-free (SPF), male BALB/c mice were purchased from the Animal Experimental Centre of the 2nd Affiliated Hospital of Harbin Medical University (Harbin, Heilongjiang, China) and housed in a controlled-environment animal facility at 22 ± 2 °C with a 12-h light/dark cycle. The animals were allowed to acclimate for 1 week prior to experiments and then provided a HFD (45% of calories from fat; Research Diet D12451) and water *ad libitum* for 10 weeks to induce obesity. The normal control group received a standard chow diet (CD, 10% of calories from fat; Research Diet D12450B). The HFD and CD were isocaloric. The mice were divided into two groups of HFD-induced obesity mice (HIO) or non-obesity mice (NO) after 10 weeks HFD feeding according to body weight of 20% higher or less than 10% difference from that of the CD group, respectively. After that, the HIO and NO mice were continued to be fed with HFD for 10 weeks. The HIO or SCFAs group was fed an HFD diet for extended 10 weeks and simultaneously administered saline or SCFA mix, composed with 67.5 mM acetate, 40 mM butyrate, and 25.9 mM propionate, by gavage at 300 mmol/L every day, respectively. Control mice received normal saline intragastrically. The body weight was recorded every 3 days during the study. Blood was collected from the retro orbital socket prior to sacrifice. Adipose and visceral tissues, as well as the femur, were obtained, weighed, snap frozen and stored at −80 °C until use. The fresh feces were stored at −80 °C for high-throughput sequencing for bacteria and metabolomics analysis.

### Fecal transplantation

Feces were freshly collected from donor mice of CD, HIO, and NO group daily for 10 weeks. 10 weeks CD fed mice which received feces from donor CD group was grouped as CD^+FMT^. The recipient obese mice of HIO^+FMT^ or NO^+FMT^ were received feces from HIO or NO group, respectively, along with continued HFD diet. The donor mice in each group were allowed to freely defecate between 8 and 10 in the morning to get the mixed feces daily, and 100 mg of feces was resuspended in 1 ml of sterile saline. The solution was vigorously mixed for 10 s with a benchtop vortex (XH-5, Hangzhou Yuexin Company, China) before centrifugation at 800 *g* for 3 min. The supernatant was collected and used as transplant material. Fresh transplant material was prepared on the day of transplantation within 10 min before oral gavage to prevent changes in the bacterial composition. Male recipient mice were fed a CD or HFD were inoculated daily with fresh transplant material using oral gavage, for 10 weeks.

### Biochemical analyses

Serum was isolated by centrifugation (2000*g*, 15 min) and frozen at −20 °C. Analyses were performed according to the manufacturer’s protocols for serum triglyceride (TG), total cholesterol (TC), and serum alkaline phosphatase (ALP) (Nanjing Jiancheng Bioengineering Institute, China). Determination of serum tartrate-resistant acid phosphatase (TRAP) levels was carried out with commercially available assay kits (Beyotime, China). As an important indicator of bone mass, serum levels of receptor activator for nuclear factor-κ B ligand (RANKL) were assessed with an ELISA kit (Cloud-Clone Corp., Houston, USA).

### Histology and immunohistochemistry

Serial sagittal plane 5-μm sections were obtained from the whole medial compartment of a joint subjected to standard histological preparation, including decalcification, dehydration, and paraffin embedding. Bone samples were stained with HE or Masson trichrome, and subsequently evaluated using a modified Mankin scoring system (SI Appendix, Table 2). For bone immunohistochemistry, after dehydration, bone tissue slices were placed in EDTA antigen repair buffer (pH 8.0) (G1206, Servicebio, Wuhan, China) and goat serum and incubated for 30 min, followed by overnight incubation 4 °C with anti-asteoprotegerin rabbit pAb primary antibody (GB11151, Servicebio, Wuhan, China) and anti-rankl rabbit pAb (GB11235, Servicebio, Wuhan, China). Slices were then washed and incubated with corresponding Cy3 conjugated goat anti-rabbit second antibody (GB21303, Servicebio, Wuhan, China) at room temperature for 50 min. The slices were then counterstained with diamidino phenyl indole (DAPI) (G1012, Servicebio, Wuhan, China), and incubated with spontaneous fluorescence quenching reagent (G1221, Servicebio, Wuhan, China) for 5 min. After washed with running tap water for 10 min, the slices were then mounted using anti-fade mounting medium (G1401, Servicebio, Wuhan, China). Images were collected by fluorescent microscopy (DS-U3, Nikon, Tokyo, Japan). For bone immunohistochemistry, after paraffin embedding, 5-μm-thick colon tissues were repaired with an antigen repair fluid (Shunobai Biotechnology Co., Ltd., Shanghai, China) and sequentially incubated in H_2_O_2_ followed by 10% goat serum (SL038, Solarbio life sciences, Beijing, China) for 30 min for each solution. The tissue sections were next incubated with antibodies of anti-Foxp3 (wl00721, Wanleibio, Shenyang, China), anti-IL-10 (wl01124, Wanleibio, Shenyang, China), and anti-TGF beta 1 (wl02998, Wanleibio, Shenyang, China), diluted in PBS overnight at 4 °C, and then with a horseradish peroxidase-labeled secondary antibody (31460, ThermoFisher Scientific, Rockford, USA) for 30 min. After coloration with diaminobenzidine, the sections were dehydrated, cleared, sealed, and observed under a microscope (BX53, Olympus, Tokyo, Japan) and camera (DP73, Olympus, Tokyo, Japan). Results of histology staining and immunohistochemical labeling were assessed in a blinded manner by three technicians who were unaware of the findings on bone loss and inflammation.

### Evaluation of physical and biomechanical properties of the femur

The femoral breaking force was measured according to a three-point bending method using a universal material testing machine (Instron 5569, USA) at a constant load speed of 1 mm/min and span length of 6 mm. The maximum flexural stress (MPa), maximum flexural load (N), and elastic modulus (MPa) values were obtained from the curve.

### Micro-computed tomography (μ-CT)

Right femur bone samples were fixed for 48 h in 4% polyformaldehyde, and rinsed three times with PBS. Femur images were reconstructed with a SCANCO μ-CT 100 in vitro system (Hangzhou Yue Bo Biological Technology Co., China). The femur was imaged with an X-ray tube voltage of 70 kV and current of 200 μA. The femur images were analyzed with SCANCO μ-CT 100 analysis software to measure density (mg HA/ccm), total volume (TV), bone volume (BV), relative bone volume (BV/TV), trabecular number (Tb. N, n), mean trabecular thickness (Tb. Th, μm), trabecular separation/spacing (Tb. Sp), bone surface (BS) and the BS/BV ratio.

### RNA isolation and real-time PCR

Total RNA was isolated with TRNZOL-A + total RNA extraction reagent (Tian Gen Biochemical Technology Co., Ltd., Beijing, China). Equal amounts of total RNA were used to synthesize cDNA with a cDNA synthesis kit (Transgen Biotech, Beijing, China). Amplifications were performed on an ABI Prism 7300 Real-Time PCR System (Applied Biosystems, America), with an initial denaturation step at 94 °C for 30 s, followed by 40 cycles of 94 °C for 5 s and 55 °C for 30 s. Relative quantification was performed using the 2^-△△CT^ method. Data are reported as fold changes relative to the expression levels of the housekeeping gene. All the primers used in our present study are shown in Supplementary Table 2.

### High-throughput sequencing of the 16S rDNA gene V3-V4 regions of fecal bacteria

Fecal DNA was extracted using a Mag-Bind® Soil DNA Kit (OMEGA), For the first round of amplification, 16S rRNA gene V3-V4 regions were amplified using the following specific primers with barcodes: 338F (ACTCCTACGGGAGGCAGCA) and 806 R (GGACTACHVGGGTWTCTAAT). The samples were incubated with Illumina bridge PCR compatible primers for the second round of amplification. Samples were then distinguished by barcode and analyzed based on operational taxonomic unit (OTU) clustering and species classification, after controlling and filtering for the quality of the sample sequence.

### Fecal metabolomics

#### Sample preparation

The collected and frozen feces from individual animal were thawed on ice and grounded to a fine powder with liquid nitrogen. Proteins were precipitated with 50% precooled methanol under vigorous shaking for 1 min, and incubated at room temperature for 10 min, followed by overnight at -20 °C. After centrifugation at 4,000 g for 20 min, the supernatants were transferred into a 96-well plate. The samples were stored at 80 °C prior to the LC-MS analysis.

#### UPLC-MS

All samples acquired by the LC-MS system followed machine orders. All chromatographic separations were performed using an UPLC system (SCIEX, Warrington, UK). An ACQUITY UPLC T3 column (100 mm×2.1 mm, 1.8 μm, Waters, UK) was used for the reversed-phase separation with a mobile phase consisting of solvent A (water, 0.1% formic acid) and solvent B (Acetonitrile, 0.1% formic acid). The extract was gradient eluted as follows: 0-5-0.5 min, 5% B; 0.5-7 min, 5-100% B; 7-8 min, 100% B; 8-8.1 min, 100-5% B and 8.1-10 min, 5% B. A high-resolution tandem mass spectrometer (Triple TOF 5600 plus, SCIEX, Warrington, UK) was used to detect metabolites eluted from the column. The Q-TOF was operated in both positive and negative ion modes with ionspray voltage floating at 5000 V and −4500 V, respectively. Mass spectrometry data were acquired in IDA mode, in the rage from 60-1200 Da with 150 ms.

#### Data analysis

LC-MS raw data files were converted into mzXML format and then processed by the XCMS, CAMERA and metaX toolbox implemented with the R software. The online KEGG, HMDB database was used to annotate the metabolites by matching the exact molecular mass data (m/z) of samples with those from the database. The intensity of peak data was further preprocessed by metaX. The P value was adjusted for multiple tests using an FDR (Benjamini–Hochberg). Supervised PLS‐DA was conducted through metaX to discriminate the different variables between groups. The VIP value was calculated. A VIP cut‐off value of 1.0 was used to select important features.

### Targeted SCFAs measurement

Fecal SCFA contents were analyzed with a Thermo Scientific Q Exactive Focus (Wilmington, DE, USA). 1 mg of samples prepared as described above was added to 50% acetonitrile aqueous solution in the ratio of 1∶200 and centrifuged at 12000 × g for 10 min. 40 μL supernatant of the sample was mixed with 20 μL 200 mM 3-NPH (dissolved in 50% acetonitrile water) and 20 μL 120 mM EDC-6% pyridine solution (dissolved in 50% acetonitrile water). The samples were then placed in aqueous solution at 40°C for 30 min and then taken out and placed on ice for 1 min after derivatization and diluted it to 2 mL with 10% acetonitrile water. After mixing with isotope internal standard at 1∶1, sample loading was carried out.

### Flow cytometry

A single-cell suspension of mesenteric lymph nodes was obtained by grinding fresh tissue through a 70 µm cell strainer, and then gently washing the tissue with PBS. The obtained cells were washed twice and then resuspended in RPMI medium. For Treg analysis, lymphocytes (2 × 10^6^) were labeled with CD4-FITC and CD25-PE for 30 min at 4 °C and then washed with fixation-permeabilization buffers. Next, precooled PBS was added to the surface-stained cells, followed by the addition of intracellular staining permeabilization wash buffer. After centrifugation (1500 *g*), the cells were labeled intracellularly with Pacific Blue-conjugated antibodies specific for Foxp3. For assessment of CD3, 4, and 8 expression, cells were labeled with FITC anti-mouse CD3, PE anti-mouse CD4, and APC anti-mouse CD8, and incubated for 30 min at 4 °C. The cells were then resuspended in PBS. Cells were analyzed using an ACEA Novocyte^TM^ (Agilent, USA) Flow cytometry and NovoExpress software. All antibodies and buffers were purchased from Biolegend (San Diego, CA) (SI Appendix, Table 3).

### TRAP staining of RAW264.7 microphages

Mice were sacrificed and sterilized with 75% ethanol. The spleens were removed and ground to obtain the lymphocytes. The obtained lymphocytes were then added into lymphocyte separation medium and centrifuged at 1500 rpm/ min for 20 min. The supernatants were then aspirated, and monocytes were washed with PBS. The cells were resuspended in RPMI 1640 medium supplemented with 20% BSA, and then added into a nylon wool fiber column for 1-h incubation. T cells were then obtained by rinsing with HANKS and BSA. RAW264.7 macrophages, used as a preosteoclast model, were incubated in 89% DMEM medium until they reached 80% confluence, and were then cocultured for 10 days with T cells extracted from the treated mice. The stimulated RAW264. 7 macrophages were stained using a TRAP kit (Beijing Leagene biotech, Beijing, China). Treatment with 100 ng/mL RANK added into the cell culture medium, was used as positive control. In contrast, DMEM medium without RANKL was used to culture negative-control cells.

### Reporting summary

Further information on research design is available in the Nature Research Reporting Summary linked to this article.

## Supplementary information

supplemental figures and tables

Reporting Summary

## Data Availability

The data that support the findings of this study are openly available in the MetaboLights database for the metabolomics mass spectrometry data with the URL http://www.ebi.ac.uk/metabolights/MTBLS5432, reference number MTBLS5432, 16S rDNA gene sequencing data were deposited in the SRA repository and can be accessible with the link: https://www.ncbi.nlm.nih.gov/sra/PRJNA882941, reference number PRJNA882941.
