# Supplementary material for: Obesity, but not high-fat diet, is associated with bone loss that is reversed via CD4+CD25+Foxp3+ Tregs-mediated gut microbiome of non-obese mice
Source: NPJ Sci Food. 2023 Apr 13;7:14. doi: 10.1038/s41538-023-00190-6 (PMC10102288; doi:10.1038/s41538-023-00190-6)
Supplement: Supplementary file 1 — supplemental figures and tables [file 41538_2023_190_MOESM1_ESM.pdf]

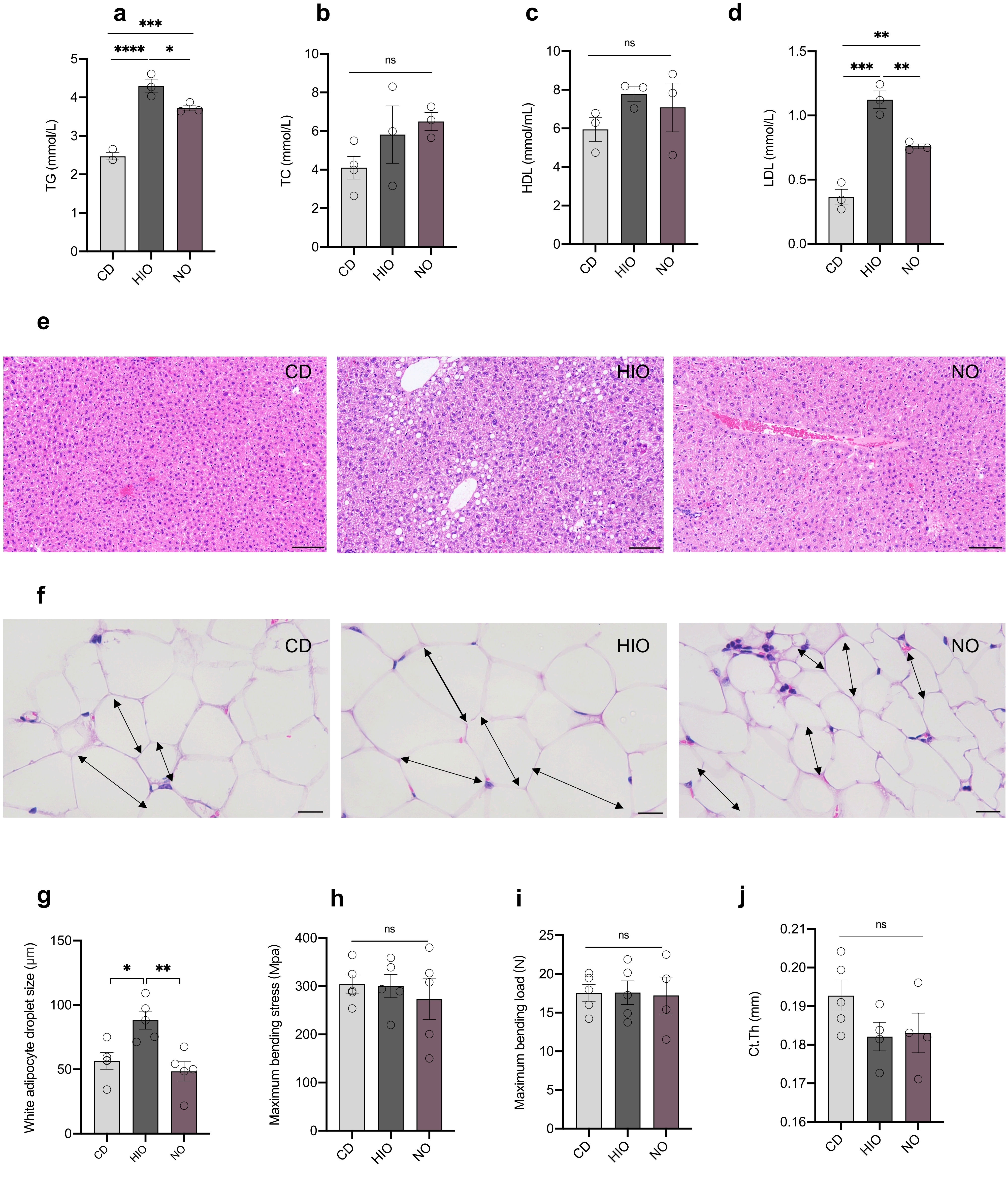

Figure. S1 NO ameliorate HFD induced bone loss. Related to Figure.1. a-d, (a) serum TG, (b) TC, (c) HDL and (d) LDL of CD, HIO and NO mice after 20 weeks feeding with chow diet or HFD respectively (n=3-4). **e,** HE staining for liver tissue, length of the scale bar is 100 μm. **f,** HE staining for white adipose tissue, length of the scale bar is 20 μm. **g,** lipid droplet size of white adipose tissue. **h-i**, (h) a three-point bending test for biomechanical analysis of tibia on maximum bending stress and (i) maximum bending load (n=4-5). **j**, cortical bone microarchitecture of tibia showing DT-Ct.Th^{+} (n=4-5). One-way ANOVA was used to assess statistical differences. $^{*}p<0.05$, $^{**}p<0.01$, $^{***}p<0.001$, $^{****}p<0.0001$.

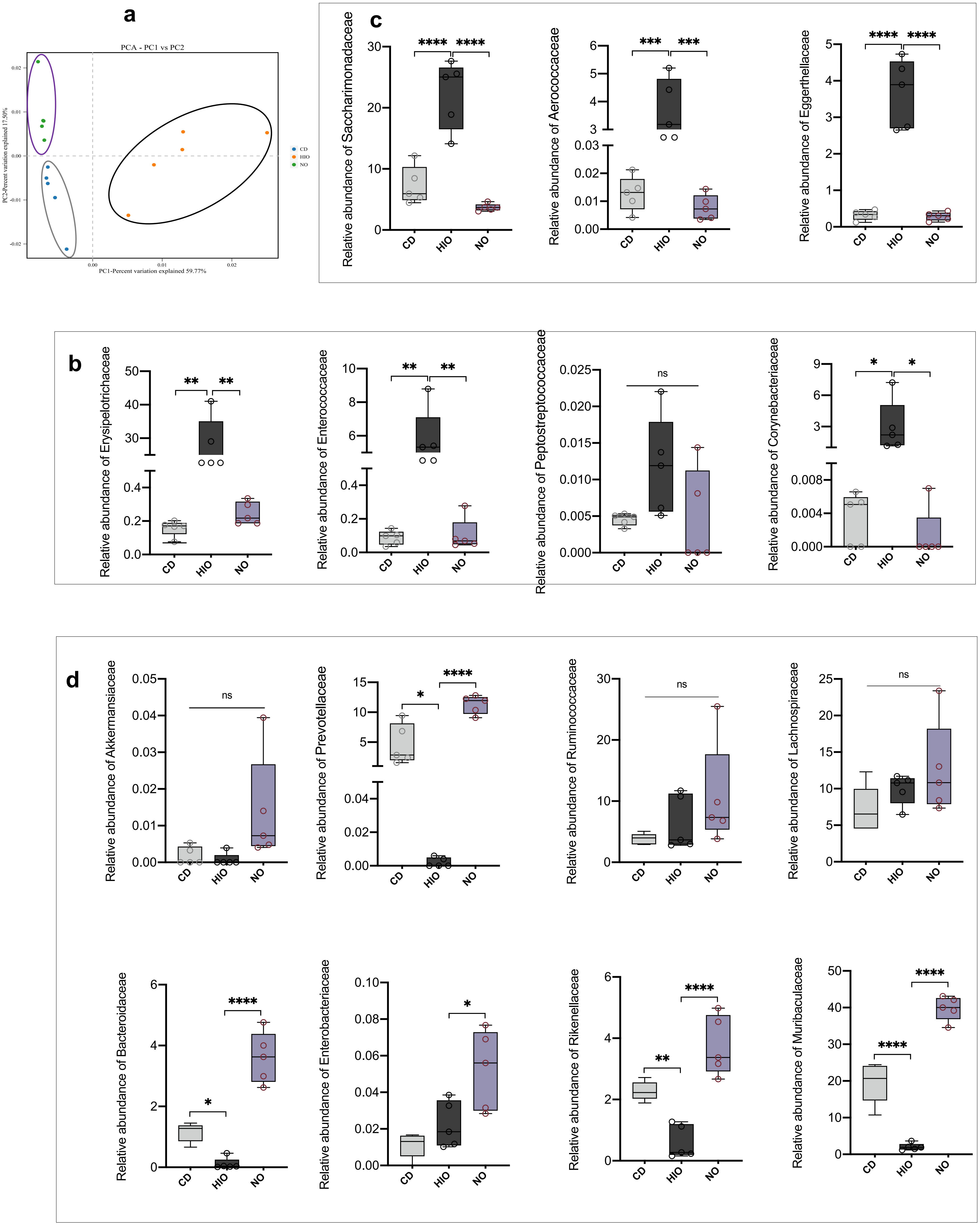
a
PCA - PC1 vs PC2
PC1-Percent variation explained 59.77%
PC2-Percent variation explained 17.50%
CD
HIO
NO
b
Relative abundance of Erysipelotrichaceae
Relative abundance of Enterococcaceae
Relative abundance of Peptostreptococcaceae
Relative abundance of Corynebacteriaceae
c
Relative abundance of Saccharimonadaceae
Relative abundance of Aerococcaceae
Relative abundance of Eggerthellaceae
d
Relative abundance of Akkermansiaceae
Relative abundance of Prevotellaceae
Relative abundance of Ruminococcaceae
Relative abundance of Lachnospiraceae
Relative abundance of Bacteroidaceae
Relative abundance of Enterobacteriaceae
Relative abundance of Rikenellaceae
Relative abundance of Muribaculaceae
ns

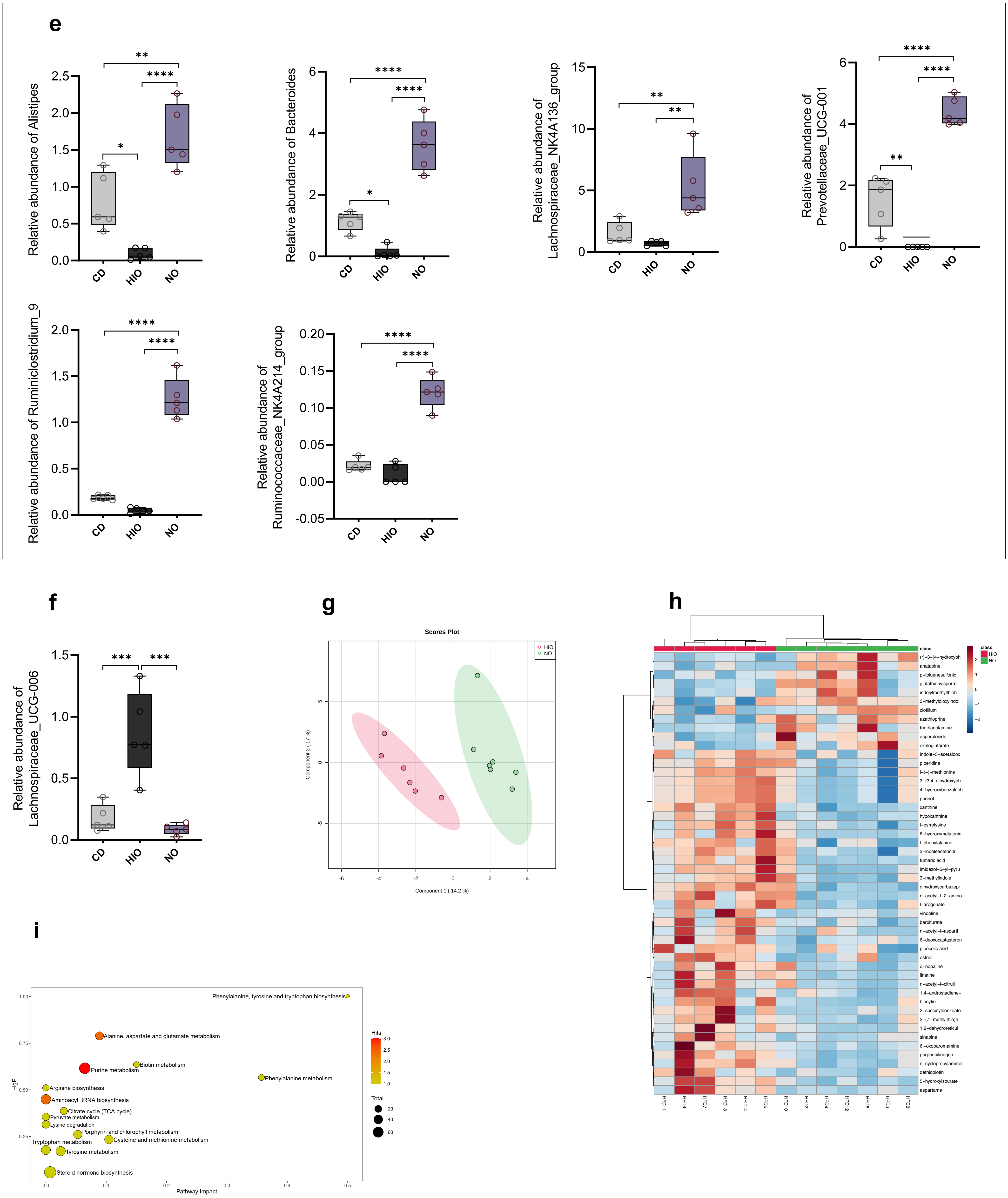

e
Relative abundance of Alistipes
Relative abundance of Bacteroides
Relative abundance of Lachnospiraceae_NK4A136_group
Relative abundance of Prevotellaceae_UCG-001
Relative abundance of Ruminiclostridium_9
Relative abundance of Ruminococcaceae_NK4A214_group
CD
HIO
NO
f
Relative abundance of Lachnospiraceae_UCG-006
g
Scores Plot
HIO
NO
Component 1 (14.2 %)
Component 2 (17 %)
h
class
i
Phenylalanine, tyrosine and tryptophan biosynthesis
Alanine, aspartate and glutamate metabolism
Biotin metabolism
Purine metabolism
Phenylalanine metabolism
Arginine biosynthesis
Aminoacyl−tRNA biosynthesis
Citrate cycle (TCA cycle)
Pyruvate metabolism
Lysine degradation
Porphyrin and chlorophyll metabolism
Tryptophan metabolism
Cysteine and methionine metabolism
Tyrosine metabolism
Steroid hormone biosynthesis
Hits
Total
−lgP
Pathway Impact

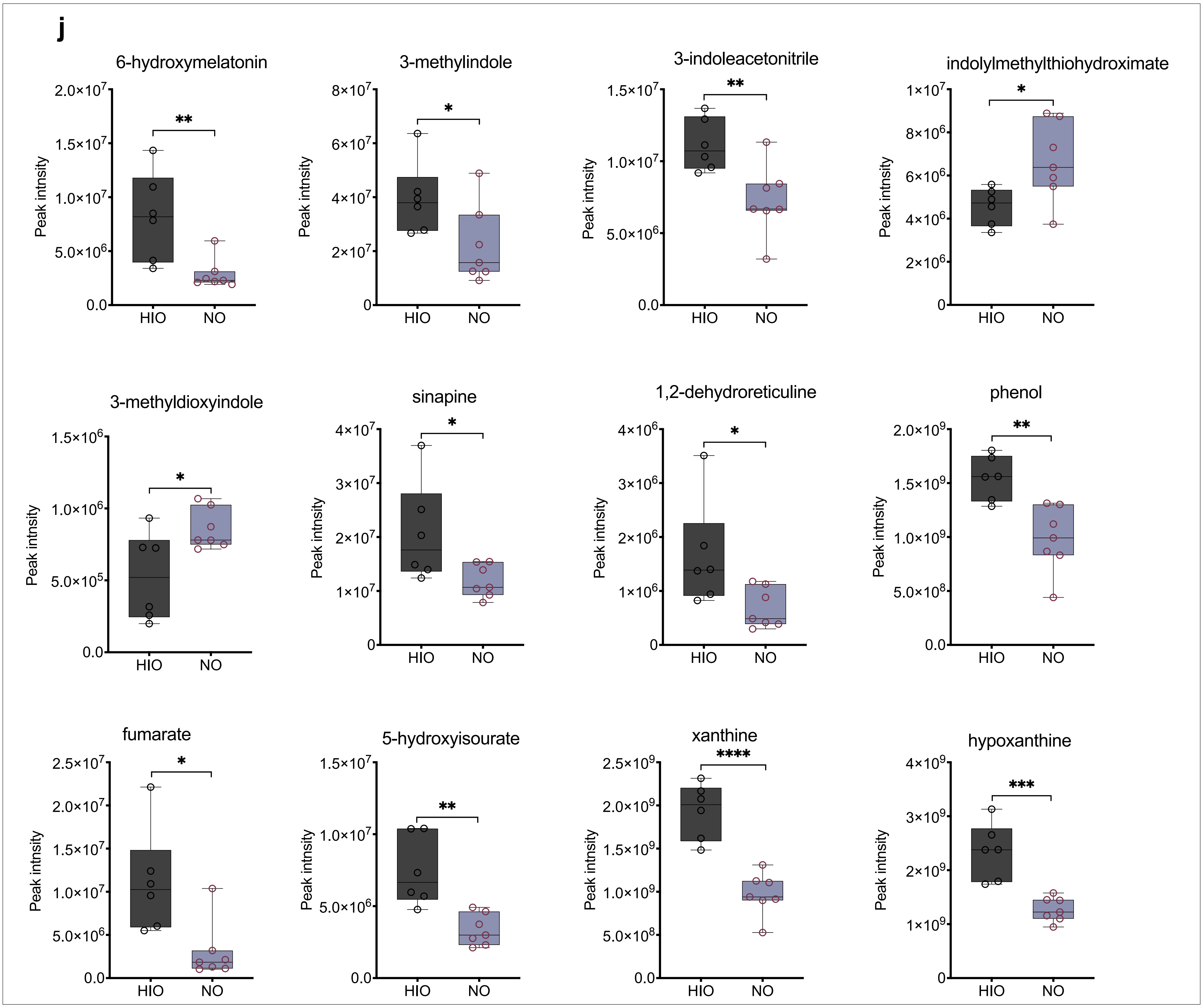
j
6-hydroxymelatonin
3-methylindole
3-indoleacetonitrile
indolylmethylthiohydroximate
3-methyldioxyindole
sinapine
1,2-dehydroreticuline
phenol
fumarate
5-hydroxyisourate
xanthine
hypoxanthine
Peak intnsity
HIO
NO

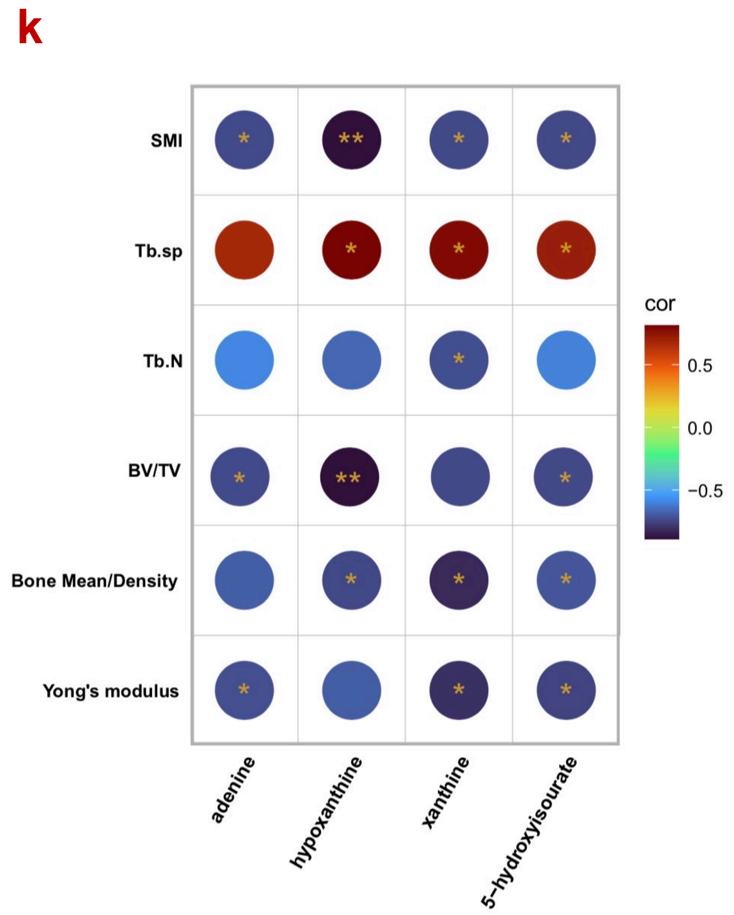
k
SMI
Tb.sp
Tb.N
BV/TV
Bone Mean/Density
Yong's modulus
adenine
hypoxanthine
xanthine
5-hydroxyisourate
cor
0.5
0.0
−0.5

Figure. S2 HIO and NO mice shows different gut microbiota and accompanying metabolites. Related to Figure.2. a, PCA plot based on Unweighted_Unifrac distances of gut microbiota composition. **b**, relative abundance of *Erysipelotrichaceae*, *Enterococcaceae*, *Peptostreptococcaceae* and *Corynebacteriaceae*. **c**, relative abundance of *Saccharimonadaceae*, *Aerococcaceae*, and *Eggerthellaceae*. **d**, relative abundance of *Akkemansiaceae*, *Prevotellaceae*, *Ruminococcaceae*, *Lachnospiraceae*, *Bacteroidaceae*, *Enterobacteriaceae*, *Rikenellaceae* and *Muribaculaceae.* **e**, relative abundance of *Alistipes*, *Bacteroides*, *Lachnospiraceae_NK4A136_group*, prevotellaceae_UCG-001, *Ruminiclostridium_9* or *Ruminococcaceae_NK4A214_group*. **f**, relative abundance of *Lachnospiraceae_UCG-006*. **g**, PCA score plot of the metabolites. **h**, heatmap displaying differential metabolites between HIO and NO. **i**, pathway impact of metabolic pathways. **j**, peak intensity of 6-hydroxymelatonin, 3-methylindole pyruvate, 3-indoleacetonitrile, indolylmethylthiohydroximate, 3-methyldioxyindole, 5-Hydroxyisourate, Xanthine, and Hypoxanthine. **k**, correlations between four types of purines and bone property parameters in HIO and NO groups using Pearson test by "R" packet. n=5 per group for high-throughput sequencing of the 16S rDNA gene of fecal bacteria; n=5-7 per group for Fecal metabolomics. Significant differences between values were evaluated using one-way ANOVA. $^{*}p<0.05$, $^{**}p<0.01$, $^{***}p<0.001$, $^{****}p<0.0001$.

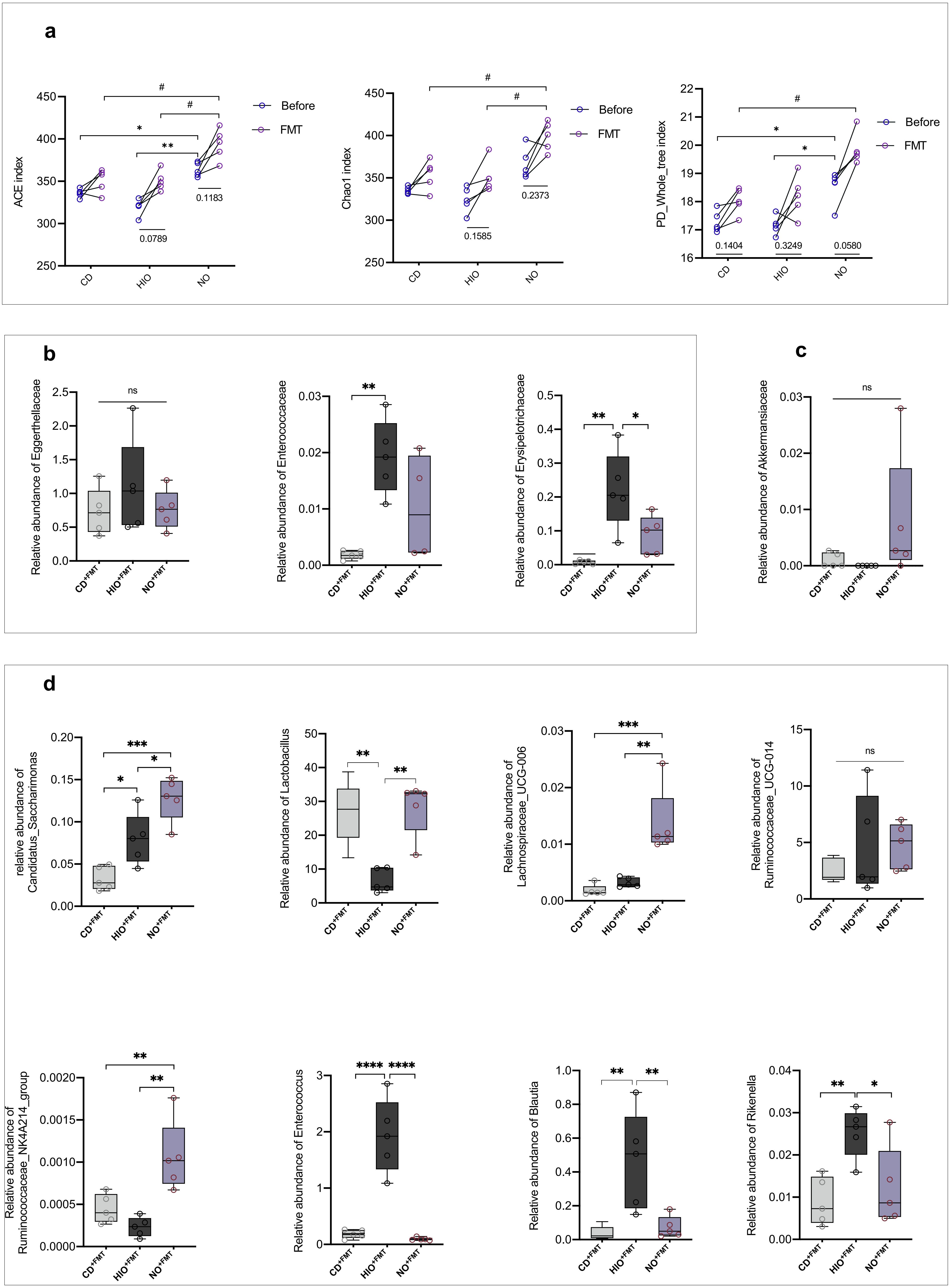

a
ACE index
Chao1 index
PD_Whole_tree index
Before
FMT
CD
HIO
NO
0.0789
0.1183
0.1585
0.2373
0.1404
0.3249
0.0580
b
Relative abundance of Eggerthellaceae
Relative abundance of Enterococcaceae
Relative abundance of Erysipelotrichaceae
c
Relative abundance of Akkermansiaceae
ns
d
relative abundance of Candidatus_Saccharimonas
Relative abundance of Lactobacillus
Relative abundance of Lachnospiraceae_UCG-006
Relative abundance of Ruminococcaceae_UCG-014
Relative abundance of Ruminococcaceae_NK4A214_group
Relative abundance of Enterococcus
Relative abundance of Blautia
Relative abundance of Rikenella
CD+FMT
HIO+FMT
NO+FMT

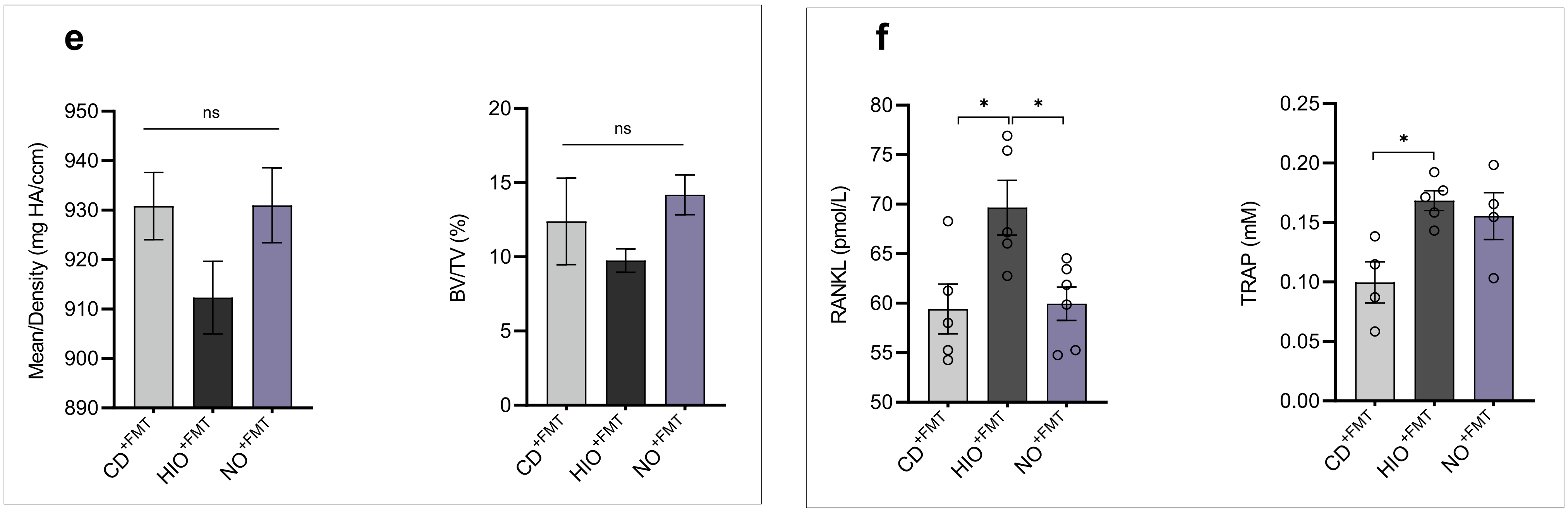

Figure. S3 NO microbiota transplantation ameliorates gut dysbiosis and prevents bone loss. Related to Figure.3. a, ACE index, Chao 1 index and PD_Whole_tree index of fecal microbiota of mice before and after transplantation. **b-d**, representing relative abundance of the most changed OTUs related to SCFAs or intestinal inflammation including (b) *Eggerthellaceae*, *Enterococcaceae*, *Erysipelotrichaceae*, and (c) *Akkemansiaceae*, and (d) *Candidatus_Saccharimonas*, *Lactobacillus*, *Lachnospiraceae_UCG-006*, *Ruminococcaceae_UCG-014*, *Ruminococcaceae_NK4A214_group*, *Enterococcus*, Blautia and *Rikenella*. **e**, femur bone mean/density and BV/TV detected by μ-CT. **f**, (r)serum content of RANKL, TRAP and ALP. n=5 per group for high-throughput sequencing of the 16S rDNA gene of fecal bacteria; n=3 per group for μ-CT; n=4-6 for osteoclast associated cytokines. Significant differences between values were evaluated using one-way ANOVA. $^{*}p<0.05$, $^{**}p<0.01$, $^{***}p<0.001$, $^{****}p<0.0001$.

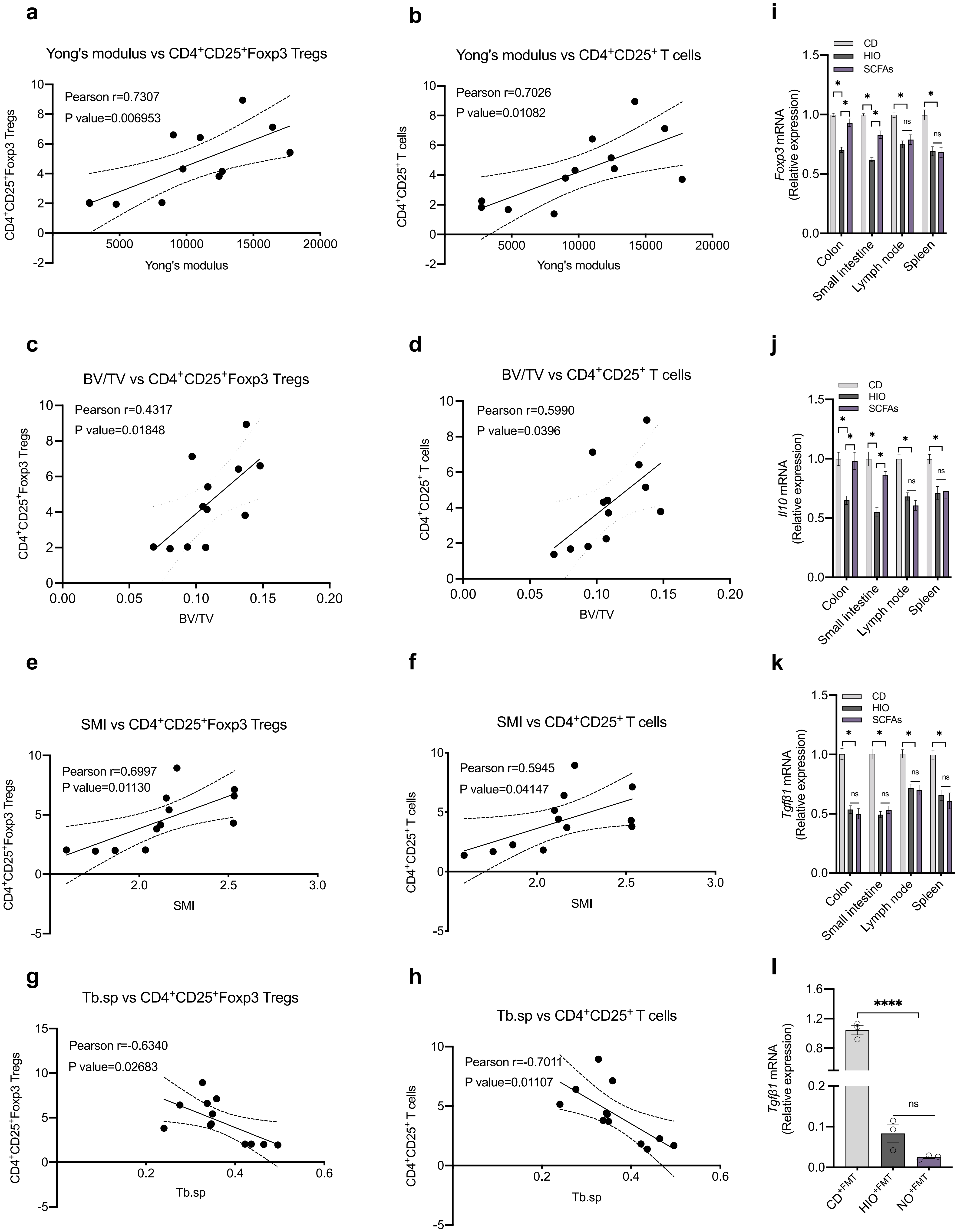

a
Yong's modulus vs CD4+CD25+Foxp3 Tregs
Pearson r=0.7307
P value=0.006953
CD4+CD25+Foxp3 Tregs
Yong's modulus
b
Yong's modulus vs CD4+CD25+ T cells
Pearson r=0.7026
P value=0.01082
CD4+CD25+ T cells
Yong's modulus
i
CD
HIO
SCFAs
Foxp3 mRNA (Relative expression)
ns
Colon
Small intestine
Lymph node
Spleen
c
BV/TV vs CD4+CD25+Foxp3 Tregs
Pearson r=0.4317
P value=0.01848
CD4+CD25+Foxp3 Tregs
BV/TV
d
BV/TV vs CD4+CD25+ T cells
Pearson r=0.5990
P value=0.0396
CD4+CD25+ T cells
BV/TV
j
CD
HIO
SCFAs
Il10 mRNA (Relative expression)
ns
Colon
Small intestine
Lymph node
Spleen
e
SMI vs CD4+CD25+Foxp3 Tregs
Pearson r=0.6997
P value=0.01130
CD4+CD25+Foxp3 Tregs
SMI
f
SMI vs CD4+CD25+ T cells
Pearson r=0.5945
P value=0.04147
CD4+CD25+ T cells
SMI
k
CD
HIO
SCFAs
Tgfβ1 mRNA (Relative expression)
ns
Colon
Small intestine
Lymph node
Spleen
g
Tb.sp vs CD4+CD25+Foxp3 Tregs
Pearson r=-0.6340
P value=0.02683
CD4+CD25+Foxp3 Tregs
Tb.sp
h
Tb.sp vs CD4+CD25+ T cells
Pearson r=-0.7011
P value=0.01107
CD4+CD25+ T cells
Tb.sp
l
Tgfβ1 mRNA (Relative expression)
****
ns
CD+FMT
HIO+FMT
NO+FMT

Figure.S4 Tregs was involved in bone loss amelioration by NO. Related to Figure.4. **a-b**, correlation between (a) $CD4^+CD25^+$ Foxp3 Tregs and (b) $CD4^+CD25^+$ T cells (*y axis*) against the Young's modules (*x axis*). **c-d**, correlation between (c) $CD4^+CD25^+$ Foxp3 Tregs and (d) $CD4^+CD25^+$ T cells (*y axis*) against BV/TV of cancellous (*x axis*). **e-f**, correlation between (e) $CD4^+CD25^+$ Foxp3 Tregs and (f) $CD4^+CD25^+$ T cells (*y axis*) against SMI of trabecula (*x axis*). **g-h**, correlation between (g) $CD4^+CD25^+$ Foxp3 Tregs and (h) $CD4^+CD25^+$ T cells (*y axis*) against Tb.sp of trabecula (*x axis*). **i-k**, mRNA expression of Foxp3 Tregs relevant factors including (i) Foxp3, (j) IL-10 and (k) TGF-β1 (n=3 per group). **l**, mRNA expression of TGF-β1 after fecal transplantation (n=3 per group). Correlations were performed using Pearson test between two different locations of recipients and kinds of genus by "R" packet. Significant differences between values were evaluated using two-way ANOVA for i-k or one-way ANOVA for l. $^*p<0.05$, $^{**}p<0.01$, $^{***}p<0.001$, $^{****}p<0.0001$.

Supplementary

Table 1. Mankin score

Parameters	Description	Score
Structure	Normal	0
	Surface irregularities	1
	Pannus and surface irregularities	2
	Clefts to the transitional zone	3
	Clefts to the calcified zone	4
	Complete lack of cartilage	5
Cellularity	Normal	0
	Diffuse hypercellularity	1
	Clusters	2
	Hypocellularity	3
Matrix staining	Normal	0
	Slight destruction	1
	Moderate destruction	2
	Severe destruction	3
	No staining or denudation	4
Tidemark integrity	Intact	0
	Destroyed	1

Table 2. Gene primer sequences

Gene		Primer sequence (5'-3')
RANKL	F	5'- TCACTCTGTCCTCTTGGTA -3'
	R	5'- CGCTTCCCGATGTTTCA -3'
OPG	F	5'- GAGAGTGAGGCAGGCTAT -3'
	R	5'- TGTGAGGAGAGGAAGGAAG -3'
TRAP	F	5'- CACTCCCACCCTGAGATTTGT -3'
	R	5'- CATCGTCTGCACGGTTCTG -3'
Col1α1	F	5'- ACGTCCTGGTGAAGTTGGTC -3'
	R	5'- TCCAGCAATACCCTGAGGTC -3'
CTSK	F	5'- TTGTGACCGTGATAATGTGA -3'
	R	5'- GCAGGCGTTGTTCTTATTC -3'
Foxp3	F	5'- GCAGAGAGGTATTGAGGGTGG -3'
	R	5'- CCACAGCATGGGTCTGTCTTC -3'
IL-10	F	5'- CCAAGCCTTATCGGAAATGA -3'
	R	5'- TCCTGAGGGTCTTCAGCTTC -3'
TGF-β	F	5'- CAATTCCTGGCGTTACCTTG -3'
	R	5'- AGCCCTGTATTCCGTCTCCT -3'
Ffar2	F	5'- GGCTTCTACAGCAGCATCTA -3'
	R	5'- AAGCACACCAGGAAATTAAG -3'
HDAC1	F	5'-CTGCGTTCTATTCGCCCAGA-3'
	R	5'-TTCACAGCACTTGCGACAGA-3'
HDAC2	F	5'-CTATCCCGCTCTGTGCCCTA-3'
	R	5'-CCTCCTTGACTGTACGCCAT-3'
HDAC7	F	5'-CTTTCTCAGGCTGCTCTCCC -3'
	R	5'-AGGGCTATGGAGGTGCATCA -3'
P300	F	5'- ACCACCACCAGCAACA -3'
	R	5'- AAGGAGCAGCAGGAAGT -3'
CREBBP	F	5'- CTGAGATGATGGAAGAGGATT -3'
	R	5'- GAAGGAGATGTTGATTGTGAG -3'

Gene		Primer sequence (5’-3’)
PCAF	F	5’- ATGAGTCCTGAAATGGCAGAGG -3’
	R	5’- CAAGTGAGAAACGTGAGCAGC -3’

Table 3. Antibodies and buffers used in flow cytometry

Item No.	Antibodies and buffers		Company
421002	Intracellular Staining Permeabilization Wash Buffer (10X)	100 mL	Biolegend
420801	Fixation Buffer	100 mL	Biolegend
424401	True-Nuclear™ Transcription Factor Buffer Set	120 tests	Biolegend
100510	FITC anti-mouse CD4	500 μg	Biolegend
101903	PE anti-mouse CD25	25 μg	Biolegend
126407	Pacific Blue anti-mouse FOXP3	25 μg	Biolegend
100306	FITC anti-mouse CD3	500 μg	Biolegend
100408	PE anti-mouse CD4	200 μg	Biolegend
100712	APC anti-mouse CD8α	100 μg	Biolegend
100510	FITC anti-mouse CD4	500 μg	Biolegend
101903	PE anti-mouse CD25	25 μg	Biolegend

Gate strategy example

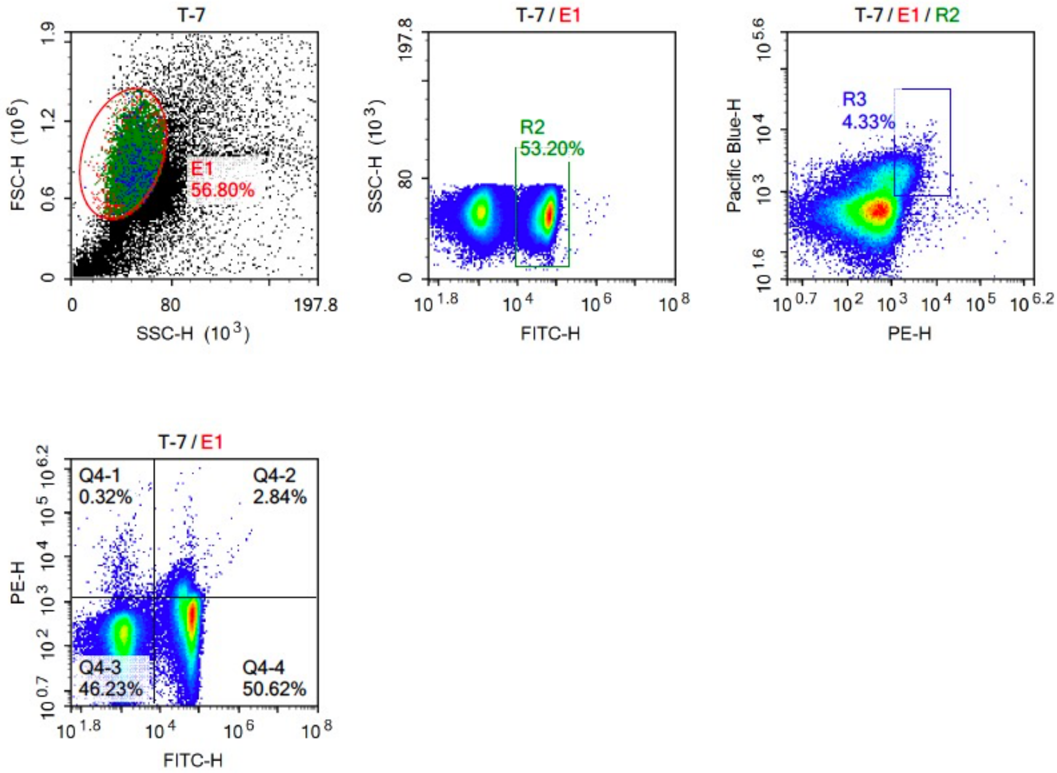

Gate	Count	% Parent	X	Y	Mean X	Mean Y	Median X	Median Y
All	269,827							
└ E1	153,261	56.80%	SSC-H	FSC-H	51,437	829,755	51,671	812,301
├ R2	81,536	53.20%	FITC-H	SSC-H	55,835	50,784	57,343	50,832
└ R3	3,531	4.33%	PE-H	Pacific Blue-H	2,075	2,045	1,704	1,813
├ Q4-1	488	0.32%	FITC-H	PE-H	1,761	17,861	1,169	3,675
├ Q4-2	4,348	2.84%	FITC-H	PE-H	48,065	9,924	39,366	1,699
├ Q4-3	70,851	46.23%	FITC-H	PE-H	1,261	50	1,106	45
└ Q4-4	77,574	50.62%	FITC-H	PE-H	56,259	178	57,831	196
