# Supplementary material for: Obesity, but not high-fat diet, is associated with bone loss that is reversed via CD4+CD25+Foxp3+ Tregs-mediated gut microbiome of non-obese mice
Source: NPJ Sci Food. 2023 Apr 13;7:14. doi: 10.1038/s41538-023-00190-6 (PMC10102288; doi:10.1038/s41538-023-00190-6)
Supplement: Supplementary file 2 — Reporting Summary [file 41538_2023_190_MOESM2_ESM.pdf]

Corresponding author(s): Wei Song, Tianli Yue

Last updated by author(s): Feb 20, 2023

Reporting Summary

Nature Portfolio wishes to improve the reproducibility of the work that we publish. This form provides structure for consistency and transparency in reporting. For further information on Nature Portfolio policies, see our Editorial Policies and the Editorial Policy Checklist.

Statistics

For all statistical analyses, confirm that the following items are present in the figure legend, table legend, main text, or Methods section.

n/a	Confirmed	
☐	☒	The exact sample size (*n*) for each experimental group/condition, given as a discrete number and unit of measurement
☐	☒	A statement on whether measurements were taken from distinct samples or whether the same sample was measured repeatedly
☐	☒	The statistical test(s) used AND whether they are one- or two-sided<br>*Only common tests should be described solely by name; describe more complex techniques in the Methods section.*
☐	☒	A description of all covariates tested
☐	☒	A description of any assumptions or corrections, such as tests of normality and adjustment for multiple comparisons
☐	☒	A full description of the statistical parameters including central tendency (e.g. means) or other basic estimates (e.g. regression coefficient) AND variation (e.g. standard deviation) or associated estimates of uncertainty (e.g. confidence intervals)
☐	☒	For null hypothesis testing, the test statistic (e.g. *F*, *t*, *r*) with confidence intervals, effect sizes, degrees of freedom and *P* value noted<br>*Give P values as exact values whenever suitable.*
☐	☒	For Bayesian analysis, information on the choice of priors and Markov chain Monte Carlo settings
☐	☒	For hierarchical and complex designs, identification of the appropriate level for tests and full reporting of outcomes
☐	☒	Estimates of effect sizes (e.g. Cohen's *d*, Pearson's *r*), indicating how they were calculated

Our web collection on statistics for biologists contains articles on many of the points above.

Software and code

Policy information about availability of computer code

Data collection	no software was used
Data analysis	Prism 9 for macOS version 9.0.1(128)

For manuscripts utilizing custom algorithms or software that are central to the research but not yet described in published literature, software must be made available to editors and reviewers. We strongly encourage code deposition in a community repository (e.g. GitHub). See the Nature Portfolio guidelines for submitting code & software for further information.

Data

Policy information about availability of data

All manuscripts must include a data availability statement. This statement should provide the following information, where applicable:

- Accession codes, unique identifiers, or web links for publicly available datasets
- A description of any restrictions on data availability
- For clinical datasets or third party data, please ensure that the statement adheres to our policy

The data that support the findings of this study are openly available in the MetaboLights database for the metabolomics mass spectrometry data with the URL http://www.ebi.ac.uk/metabolights/MTBLS5432, reference number MTBLS5432, 16S rDNA gene sequencing data was deposited in the SRA repository and can be accessible with the link: https://www.ncbi.nlm.nih.gov/sra/PRJNA882941, reference number PRJNA882941.

Human research participants

Policy information about studies involving human research participants and Sex and Gender in Research.

Reporting on sex and gender	This information has not been collected.
Population characteristics	*Describe the covariate-relevant population characteristics of the human research participants (e.g. age, genotypic information, past and current diagnosis and treatment categories). If you filled out the behavioural & social sciences study design questions and have nothing to add here, write "See above."*
Recruitment	*Describe how participants were recruited. Outline any potential self-selection bias or other biases that may be present and how these are likely to impact results.*
Ethics oversight	*Identify the organization(s) that approved the study protocol.*

Note that full information on the approval of the study protocol must also be provided in the manuscript.

Field-specific reporting

Please select the one below that is the best fit for your research. If you are not sure, read the appropriate sections before making your selection.

☒ Life sciences ☐ Behavioural & social sciences ☐ Ecological, evolutionary & environmental sciences

For a reference copy of the document with all sections, see nature.com/documents/nr-reporting-summary-flat.pdf

Life sciences study design

All studies must disclose on these points even when the disclosure is negative.

Sample size	Sample size was determined by the Resource Equation Approach which is as sample size =(10~20)/k+1 for group comparison by One-way ANOVA. The sample size was calculated as (10~20)/3+1=5~7 for each group. The total sample size for 3 groups were 15~21. Considering that there were several parameters that we need to determine, we choose 8-10 mice for each group.
Data exclusions	Data were excluded when they exceeded the value of mean plus or minus 3 times of standard deviation.
Replication	All experiments were performed in triplicate and were repeated at least three times to verify the reproducibility of the experimental findings. All attempts at replication were successful.
Randomization	The mice were randomly grouped as chow diet and high fat diet. The high fat-induced obesity mice (HIO) and non-obesity mice (NO) have been selected according to body weight of 20% higher or less than 10% difference from that of the CD group respectively after 10 weeks HFD feeding. Feces were freshly collected from donor mice of CD, HIO and NO group daily for extending 10 weeks. 10 weeks CD fed mice which received feces from donor CD group was grouped as CD+FMT. The recipient obese mice of HIO+FMT or NO+FMT were received feces from HIO or NO group respectively along with continued HFD diet.
Blinding	The investigators were blinded to group allocation during data collection or analysis.

Reporting for specific materials, systems and methods

We require information from authors about some types of materials, experimental systems and methods used in many studies. Here, indicate whether each material, system or method listed is relevant to your study. If you are not sure if a list item applies to your research, read the appropriate section before selecting a response.

Materials & experimental systems

n/a	Involved in the study	
☐	☒	Antibodies
☐	☒	Eukaryotic cell lines
☒	☐	Palaeontology and archaeology
☐	☒	Animals and other organisms
☒	☐	Clinical data
☒	☐	Dual use research of concern

Methods

n/a	Involved in the study	
☒	☐	ChIP-seq
☐	☒	Flow cytometry
☒	☐	MRI-based neuroimaging

Antibodies

Antibodies used

Item No. Antibodies Company
100510 FITC anti-mouse CD4 500 µg Biolegend
101903 PE anti-mouse CD25 25 µg Biolegend
126407 Pacific Blue anti-mouse FOXP3 25 µg Biolegend
100306 FITC anti-mouse CD3 500 µg Biolegend
100408 PE anti-mouse CD4 200 µg Biolegend
100712 APC anti-mouse CD8a 100 µg Biolegend
100510 FITC anti-mouse CD4 500 µg Biolegend
101903 PE anti-mouse CD25 25 µg Biolegend

Validation

Item No. Antibodies Manufacturer's website Company
100510 FITC anti-mouse CD4 https://www.biolegend.com/en-us/products/fitc-anti-mouse-cd4-antibody-480 Biolegend
101903 PE anti-mouse CD25 https://www.biolegend.com/en-us/products/pe-anti-mouse-cd25-antibody-129 Biolegend
126407 Pacific Blue anti-mouse FOXP3 https://www.biolegend.com/en-us/products/alexa-fluor-647-anti-mouse-foxp3-antibody-4662 Biolegend
100306 FITC anti-mouse CD3 https://www.biolegend.com/en-us/products/fitc-anti-mouse-cd3epsilon-antibody-23 Biolegend
100408 PE anti-mouse CD4 https://www.biolegend.com/en-us/products/pe-anti-mouse-cd4-antibody-250 Biolegend
100712 APC anti-mouse CD8 α https://www.biolegend.com/en-us/products/apc-anti-mouse-cd8a-antibody-150 Biolegend
100510 FITC anti-mouse CD4 https://www.biolegend.com/en-us/products/fitc-anti-mouse-cd4-antibody-480 Biolegend
101903 PE anti-mouse CD25 https://www.biolegend.com/en-us/products/pe-anti-mouse-cd25-antibody-129 Biolegend

Eukaryotic cell lines

Policy information about cell lines and Sex and Gender in Research

Cell line source(s): Cell line: RAW264.7; Cell: lymphocyte from spleen of male BALB/c mouse

Authentication: None of the cell lines used were authenticated.

Mycoplasma contamination: Cell lines were tested negative for mycoplasma contamination.

Commonly misidentified lines (See ICLAC register): No misidentified lines.

Animals and other research organisms

Policy information about studies involving animals; ARRIVE guidelines recommended for reporting animal research, and Sex and Gender in Research

Laboratory animals: Five-week-old, specific pathogen-free (SPF), male BALB/c mice.

Wild animals: The study did not involve the wild animals.

Reporting on sex: Male mice. Normally, the followed methods are used for bone loss or osteoporosis model including ovariectomized (OVX)/sham-operated rat model, glucocorticoid-induced osteoporosis, disuse osteoporosis, brain derived osteoporosis, gene modified osteoporosis animal model and high-fat diet induce bone loss, etc. In terms of gender selection, female rats or male rats are used for OVX model or sham rats respectively; male mice are often used for glucocorticoid-induced osteoporosis, which can avoid effect on estrogen changes induced by glucocorticoid; either gender can be used for disuse osteoporosis model, brain derived osteoporosis and gene modified osteoporosis model. According to the gender selection of the above models, we can see that the section is based on the requirement of the model itself and stability of experimental results.
Diet-induced obesity often use male mice, the reasons, we considered, are on one hand, as reviewer said males are more likely to be obesity-induced by HFD; on the other hand, females are easily to be affected by hormone, such as estrogen promotes cholesterol decomposition. Furthermore, males normally have longer femur and larger bone diameter than females based on our results, which can't be statistically analyzed together, so group composed of half males and half females is not applicable to this research. To sum up, male mice were used for HFD-induced bone loss model.

Field-collected samples: The study did not involve samples collected from the field.

Ethics oversight: Animal experimental procedures followed the National Institutes of Health guidelines for the care and use of laboratory animals and were approved by the local ethics committee (Approval No. SYXK-(HEI) 2019-001).

Note that full information on the approval of the study protocol must also be provided in the manuscript.

Flow Cytometry

Plots

Confirm that:

☒ The axis labels state the marker and fluorochrome used (e.g. CD4-FITC).

☒ The axis scales are clearly visible. Include numbers along axes only for bottom left plot of group (a 'group' is an analysis of identical markers).

☒ All plots are contour plots with outliers or pseudocolor plots.

☒ A numerical value for number of cells or percentage (with statistics) is provided.

Methodology

Sample preparation	A single-cell suspension of mesenteric lymph nodes was obtained by grinding fresh tissue through a 70 μm cell strainer, and then gently washing the tissue with PBS. The obtained cells were washed twice and then resuspended in RPMI medium.
Instrument	ACEA NovocyteTM (Agilent, USA)
Software	NovoExpress software
Cell population abundance	The purity of lymphocytes was around 20%, which was detected by flow cytometry through threshold setting to exclude the cell debris and FSC/SSC gates to find the target cells.
Gating strategy	CD4+ cells were sorted by adding FITC Fluorescence dye compared with isotype control and blank control to set the gate. The CD4+ cells were used as double negative control of CD25+Foxp3 cells to set the CD25+Foxp3 double positive gate.

☒ Tick this box to confirm that a figure exemplifying the gating strategy is provided in the Supplementary Information.
